# Supplementary material for: GRouNdGAN: GRN-guided simulation of single-cell RNA-seq data using causal generative adversarial networks
Source: Nat Commun. 2024 May 14;15:4055. doi: 10.1038/s41467-024-48516-6 (PMC11525796; doi:10.1038/s41467-024-48516-6)
Supplement: Supplementary file 1 — Supplementary Information [file 41467_2024_48516_MOESM1_ESM.pdf]

GRouNdGAN: GRN-guided simulation of single-cell RNA-seq data using causal generative adversarial networks

Yazdan Zinati[1], Abdulrahman Takiddeen[1], and Amin Emad[1,2,3,*]

[1] Department of Electrical and Computer Engineering, McGill University, Montreal, QC, Canada

[2] Mila, Quebec AI Institute, Montreal, QC, Canada

[3] The Rosalind and Morris Goodman Cancer Institute, Montreal, QC, Canada

* Corresponding Author:

Amin Emad

755 McConnell Engineering Building

3480 University Street, Montreal, Quebec, Canada, H3A 0E9

Email: amin.emad@mcgill.ca

Supplementary Notes

An ablation study for different components of GRouNdGAN

GRouNdGAN includes several components and incorporates auxiliary networks to provide a realistic simulated dataset and to ensure that the causal GRN is imposed within the generated cells. Here, we investigated the effect that these components (labeler, anti-labeler, library size normalization layer, and noise input to the target generators) have on the causal GRN enforcement and on the quality of data simulation. We conducted this ablation study on the BoneMarrow dataset. For the imposed GRN, we first identified top ten TFs for each gene using GRNBoost2 (using the real dataset), but only imposed the regulatory edge between the gene and its top 1st, 3rd, 5th, 7th, and 9th TFs. The small sample size of this dataset and the imposition of a GRN that to some degree deviates from the GRN that best matches the real reference single-cell dataset make training a model on this (dataset, GRN) combination more challenging, allowing us to observe more granularity when ablating from our model. Supplementary Table 3 shows the effect of each change in the architecture of the model on the performance. It is evident from this table that GRouNdGAN performs best across all metrics when all components are included.

The stability of GRouNdGAN and the effect of GRN properties on its performance

To assess the stability of GRouNdGAN, we generated five different batches (or “replicates”) of 1000 cells using the PBMC-CTL dataset. Comparing each generated replicate with the held-out real test set (Supplementary Data 2 - Sheet 2) revealed a stable performance across different generated replicates and a high degree of resemblance to real data. We then repeated this analysis two additional times, with different training/testing splits of the PBMC-CTL (which resulted in slightly different GRNs and required retraining of the model). Once again, not only a high degree of stability was observed within each training/testing option (across different runs or replicates), but also the results were quite consistent across training/testing options. We then repeated the analyses above using cells corresponding to CD8+/CD45RA+ Naive T cells from the same study, with n = 16,666 cells (which we called the PBMC-NaïveT dataset), which once again confirmed the observations above in a different cell type (Supplementary Data 2 - Sheet 2).

Next, we sought to determine the effect of the choice of the imposed GRN on the simulated data. We imposed different GRNs with different properties, and simulated data using the PBMC-CTL and the BoneMarrow datasets (Supplementary Data 2 - Sheets 3 and 4). We observed that increasing the number of TFs regulating each gene from 15 to 20, 25, and 30 marginally improves the resemblance of simulated data to experimental data for PBMC-CTL. On the other hand, we observed that reducing the number of TFs regulating each gene results in some deterioration of the performance. We hypothesized that this is because when we reduce the number of regulating TFs, the GRN is less representative of the TF-gene relationships of the real dataset. To test this (while controlling for the number of regulating TFs), we compared the dataset generated by a GRN composed of the top ten TFs for each gene and the dataset generated by a GRN composed of the bottom ten TFs for each gene (edges that were ranked very low by GRNBoost2 on the experimental dataset). Consistent with our hypothesis, the former dataset had much better performance (Supplementary Data 2). For example, data generated based on PBMC-CTL and top 10 TFs had an miLIS = 1.89 and MMD=0.026 (on test set), while the data generated based on bottom 10 TFs an miLIS = 1.56 and MMD=0.355.

The reason for this behavior is that when generating realistic simulated data, imposing a GRN that does not conform to the underlying TF-gene relationship in the real dataset makes the task extremely challenging. This is because incorporating the causal GRN imposes certain patterns in the gene expression profiles of samples. If this pattern is drastically different from the one present in the real dataset, generating samples resembling the training real dataset and imposing the GRN simultaneously act as contradictory requirements. This is the reason that simulators that do not impose a GRN in the data, have a much easier task, as they only need to generate realistic synthetic data even if they disrupt the TF-gene (or co-expression) relationships. In fact, we observed that when a GRN inference method (GRNBoost2) was applied to data generated by scGAN, its performance was much lower than when they were applied to real data (Figure 3), showing that some TF-gene relationships are disrupted by the simulator. In spite of this deterioration in performance, when a reasonable number of top TFs were selected (e.g., top 5 TFs), a good performance could be achieved (e.g., the test set miLISI was larger than 1.80 for both datasets).

Details regarding the Wasserstein distance

Various metrics exist to quantify the similarity between the generator's and real data's probability distributions. While initial implementations of the GAN used Kullback–Leibler or Jensen–Shannon divergence, they are prone to mode collapse where the generator learns to produce only a subset of modes in the dataset[1-4]. Numerous approaches have been proposed to solve this problem, from regularization technique to using separate generators, explicitly enforcing the GAN to learn all modes[1,4-6], etc. Wasserstein distance, when used as the divergence metric in GANs has led to stability in training without evidence of mode collapse[7]. Mathematically, the Wasserstein or Earth-Mover distance is defined as

$$W(\mathbb{P}_r, \mathbb{P}_g) = \inf_{\gamma \in \Pi(\mathbb{P}_r, \mathbb{P}_g)} \mathbb{E}_{(x,y)\sim\gamma}[\, \|x - y\| \,],$$

where $\Pi(\mathbb{P}_r, \mathbb{P}_g)$ is the set of all joint distributions over x and y whose marginals are respectively $\mathbb{P}_r$ and $\mathbb{P}_g$. Intuitively, $\gamma(x, y)$ denotes the unit of mass to be transported from x to y to transform $\mathbb{P}_r$ to $\mathbb{P}_g$ representing the optimal transport plan.

However, the infimum in equation 1 is intractable over all $\gamma \in \Pi(\mathbb{P}_\mathrm{r}, \mathbb{P}_\mathrm{g})$, for this reason an equivalent formulation of the Wasserstein distance from the Kantorovich-Rubinstein duality[8] can be obtained,

$$W(\mathbb{P}_r, \mathbb{P}_g) = \sup_{\|f\|_L \leq 1} \mathbb{E}_{x\sim\mathbb{P}_r}[f(x)] - \mathbb{E}_{x\sim\mathbb{P}_g}[f(x)],$$

where $W(\mathbb{P}_r, \mathbb{P}_g)$ is the supremum over all 1-Lipschitz functions with values in $\mathbb{R}$ ($f: X \rightarrow \mathbb{R}$). We approximated the solution f to this problem using the discriminative network (critic). To enforce a Lipschitz constraint on the critic, we added a gradient penalty term proposed by Gulrajani et al.[2] as an alternative to weight clipping used in the original WGAN which is shown to suffer from vanishing or exploding gradients and capacity underuse.

Preservation of marker genes' expression in PBMC-All

In the results reported in Table 2, we used a GRN inferred using GRNBoost2 on all cell types of the PBMC-All dataset to generate synthetic data using GRouNdGAN. This was done to show that even when cell type specific information (whether cell types or cell type-specific GRNs) are not provided to GRouNdGAN, it can still generate realistic data. Since such cell type information may not be available for some applications, this minimizes user involvement when it comes to annotating cell types or identifying cell type specific GRNs.

To better understand the effect that such a choice will have on the simulated cells and their cell types, we set out to characterize the gene expression patterns of cell type marker genes. First, we generated a synthetic dataset using GRouNdGAN with the same size as the PBMC-All training set (n = 67579 cells). Then, we trained a classifier (a C-Support Vector classifier) based on the annotated cell types of the experimental training set and used it to annotate the cell types of the synthetic datasets. Then, we generated two dot plots to visualize the expression patterns of cell type marker genes in each cell type for both the experimental and synthetic datasets (Figure S5), which showed a high degree of concordance between the two.

Simulating data using SERGIO

We used SERGIO to simulate cells from the PBMC-CTL dataset and employed the same GRN containing 15 regulating TFs per gene used to train GRouNdGAN. We computed the basal production rate b_i of each TF i from its mean expression x_i in the reference dataset, assuming a decay rate λ of 0.8:

$$b_i = \lambda \mathrm{E}[\mathrm{x_i}].$$

We followed SERGIO's approach of uniformly sampling interaction strengths parameters K_{ij} (used to compute the production rate of target genes), denoting the maximum contribution of TF j to target gene i from a range of $[-1, -5] \cup [1, 5]$. Positive and negative contribution strengths K_{ij} represent activatory and repressive interactions, respectively.

Using the simulated clean dataset, we iteratively tuned the parameters of the following modules: outlier genes, dropouts, and conversion to UMI counts. But since all cells of the reference PBMC-CTL dataset were library-size normalized to a constant of 20000, we replaced SERGIO's library size normalization module, which for each cell samples a library size from a lognormal distribution with one that assigns a library size of 20000 to all simulated cells. To compare the noise level between reference and simulated (now "noisy") datasets, we used the same statistical measures as SERGIO and fine-tuned the parameters until we did not see any improvements from one iteration to the next (Figure S4). This process took about two hours. We provided the final technical noise parameters that we found below.

Outlier Genes			Dropouts		Library Size		Cell Filtering Threshold
π^o	μ^o	σ^o	k	q	μ^L	σ^L	
0.01	0.8	1	80	95	20000	0	10

Supplementary Tables 4 and 5 summarize the test and training set performance of SERGIO before and after the described distribution matching. Overall, we observe an improvement in most metrics with the addition of technical noise to the "clean" dataset. However, even after distribution matching, SERGIO did not perform on par with other simulators that we benchmarked (see Table 1). Most likely, this is since the clean data in SERGIO is matched to the reference through five dataset-level (as opposed to gene-level or cell-level) statistics. As such, the distribution of individual genes are not matched to the reference and gene identities are not preserved.

Implementation of target generators as a large sparse network

As mentioned in Methods, implementing each target generator as a separate neural network significantly increases the computational cost and introduces excess overhead due to the LSN layer. Hence, we implemented target generators using a single large sparse network and used masks to keep them identical to the original architecture from a logical standpoint.

To achieve this, we extended PyTorch's linear module (see GRouNdGAN's API References: src/layers/masked_linear.py) to remove a neural network's edges by masking its weights and gradients. The masked linear module accepts a binary mask matrix of dimensions $(N_{Input\ neurons}, N_{Output\ neurons})$ to initialize a fully connected layer. Setting element (n, m) in the mask matrix to zero removes the edge connecting input neuron n to output neuron m by zeroing out the corresponding weights and gradients. For the target generators, we defined the following three masks:

- input mask: Defines connections between the target genes' generators and their regulating TFs and noise variables.
- hidden mask: Defines connections between the hidden layers such that there is no connection between hidden layers of two target genes' generators.
- output mask: Defines connections between the hidden layers of each gene's target generator and its outputted expression values (before the LSN layer).

The following pseudocode shows how we defined each mask matrix. In this pseudocode, the following notation is used:

- w: The width multiplier of target generator's hidden layers. As a result, the width of each target generator's hidden layer equals $w(N_{Noise} + N_{TFs\_regulating})$.
- N_{Genes}: Number of target genes in the GRN.
- N_{TFs}: Number of regulating TFs in the GRN.
- N_{Edges}: Number of edges in the imposed GRN.
- N_{Noise}: Dimension of the noise variable per target generator.

**The pseudocode for generating mask matrices**
**Start**

// Compute the combined width (hidden dimension) of all target generators' hidden layers combined ($HDim$) which equals $HDim = w(N_{Noise} \times N_{Genes} + N_{Edges})$. Each target generator's hidden dimension is $w(N_{Noise} + N_{regulating\_TFs})$. If all genes were regulated by the same number of TFs, $HDim$ would equal $N_{Genes}(w(N_{Noise} + N_{regulating\_TFs}))$. Since that is not always the case, the general formulation of HDim is equal to $HDim = w(N_{Noise} \times N_{Genes} + N_{Edges})$, where $N_{Edges} = \sum_{i=0}^{N_{Genes}} N_{regulating\_TFs\_i}$ and $N_{regulating\_TFs\_i}$ is the number of TFs regulating $Gene_i$.

// Zero initialize mask matrices of shape (rows, columns)
Input_mask = zeroes(N_{TFs}, $HDim$)
Hidden_mask = zeroes($HDim$, $HDim$)
Output_mask = zeroes($HDim$, N_{Genes})

// This variable keeps track of the width of traversed hidden dimension
Beginning_HDim = 0

// Loop through genes and for each gene, recover its index ($\mathrm{Index_{Gene}}$) and the indices of its regulating TFs ($\mathrm{Index_{regulating\_TFs}}$)
For ($\mathrm{Index_{Gene}}$, $\mathrm{Index_{regulating\_TFs}}$) in causal_graph:

    // Calculate current gene's target generator width
    Current_HDim = $w(N_{Noise} + N_{\mathrm{regulating\_TF\_indices}})$

    // unmask neural connections between regulating TFs and the input layer of the gene's target generator
    Input_mask[$\mathrm{Index_{regulating\_TFs}}$, Beginning_HDim : Beginning_HDim + Current_HDim] = 1

    // Initialize noise matrix
    // and unmask neural connections between noise inputs and the input layer of gene's target generator
    Noise_mask = zeroes(N_{Noise}, Hidden_Dimension)
    Noise_mask[: ,Beginning_HDim : Beginning_HDim + Current_HDim] = 1
    Input_mask = concatenate(Input_mask, Noise_mask) // vertically stacking (on rows dimension)

    // unmask neural connections between hidden layers of the gene's target generator
    Hidden_mask[
    Beginning_HDim : Beginning_HDim + Current_HDim,
    Beginning_HDim : Beginning_HDim + Current_HDim
    ] = 1

    // unmask neural connections between the last hidden layer and the output layer of the gene's target generator
    Output_mask[
    Beginning_HDim : Beginning_HDim + Current_HDim,
    $\mathrm{Index_{Gene}}$
    ] = 1

    Beginning_HDim += Current_HDim // Update width of traversed hidden dimension

**End For**<br>// Use the masks to initialize sparse linear layers to form the target generator.<br>**End**

Supplementary Tables

Supplementary Table 1: Architectural choices and hyperparameters of the WGAN used to pre-train the causal controller (Figure 1B).

**Hyperparameter / Architectural Choice**	**Value**
Length of the input noise vector (latent dimension)	128
Width of generator's input layer	128
Width of generator's hidden layers	[256, 512, 1024]
Width of generator's output layer	1000
Width of critic's input layer	1000
Width of critic's hidden layers	[1024, 512, 256]
Width of critic's output layer	1
Library size	20000
Regularization parameter of the gradient penalty (λ)	10
Batch size	128
Number of critic iterations per generator one iteration	5
Number of training steps	200000
AMSGrad's coefficients for computing the running averages of the gradient and its square	$\beta_1 = 0.5, \beta_2 = 0.9$
Generator's initial learning rate	0.0001
Generator's final learning rate	0.00001
Critic's initial learning rate	0.0001
Critic's final learning rate	0.00001

Supplementary Table 2: Architectural choices and hyperparameters used in GRouNdGAN (Figure 1C).

**Hyperparameter / Architectural Choice**	**Value**
Dimension of the noise variable per target generator (N_{noise})	1
Width of the input layer for the generator of a target gene with N_{TF} regulating TFs	$N_{noise} + N_{TF}$
Number of hidden layers of each target generator	3
Width multiplier (w)	2
Width of each target generator's hidden layer	$w\ (N_{noise} + N_{TF})$
Size of the output layer for each target generator	1
Width of the critic's input layer	1000
Width of critic's hidden layers	[1024, 512, 256]
Size of the critic's output layer	1
Width of the input layer for the Labeler and the Anti-Labeler	Number of target genes
Width of the hidden layers of the Labeler and the Anti-Labeler	[2000, 2000, 2000]
Width of the Labeler and the Anti-Labeler output layer	Total Number of TFs
Library size	20000
Regularization parameter of the gradient penalty (λ)	10
Batch size	1024
Number of critic iterations per generator one iteration	5
Number of training steps	700000
AMSGrad's coefficients for computing the running averages of the gradient and its square	$\beta_1 = 0.5, \beta_2 = 0.9$
Generator's initial learning rate	0.001
Generator's final learning rate	0.0001
Critic's initial learning rate	0.001
Critic's final learning rate	0.0001

Supplementary Table 3: The results of the ablation study on a held-out test set of the BoneMarrow dataset. The first column shows the variation of the model. The values in the table show the percentage of the change in the performance metrics compared to GrouNdGAN. The first five metrics quantify the resemblance of simulated and experimental data. The last column shows the decrease in the AUPRC of GRN inference using simulated data (using GRNBoost2) and quantifies the effect of ablation on GRN imposition. Note that in the last row, the normalization step and LSN layer is removed and hence the cosine distance, Euclidean distance, and MMD are not comparable between the ablated model and the original model (as they are at different scales).

Model	Increase in RF AUROC (%)	Increase in Cosine distance (%)	Increase in Euclidean distance (%)	Decrease in miLISI (%)	Increase in MMD (%)	Decrease in GRN inference AUPRC (%)
GrouNdGAN w/o labeler	16.7	4.5	7.5	40.5	16.7	5.2
GrouNdGAN w/o anti-labeler	50.0	16.7	4.0	39.3	50.0	6.5
GrouNdGAN w/o labeler & w/o anti-labeler	7.6	100.0	54.9	1.5	61.9	7.8
GrouNdGAN w/o noise to target generators	16.5	33.3	25.4	7.7	54.8	2.6
GrouNdGAN w/o LSN layer & w/o input normalization	21.5	NA	NA	-3.3	NA	5.2

Supplementary Table 4: Test set performance of SERGIO in generating realistic scRNA-seq data using the PBMC-CTL dataset. SERGIO (Clean) and SERGIO (Synth (aka noisy)) refer to the simulated data before and after distribution matching through the addition of technical noise, respectively. The metrics are calculated between a simulated dataset of 1000 cells and the held-out test set of 1000 real cells. For both SERGIO and GRouNdGAN, the same GRN was imposed. In this GRN, each gene was regulated by 15 TFs (constructed using GRNBoost2 from the experimental training set). For the first three metrics, a value closer to zero is preferred, for RF AUROC a value closer to 0.5 is preferred, and for miLISI a value closer to 2 is preferred. For the first two metrics, the values correspond to the distance of the mean centroids of the real and simulated cells. The RF AUROC of control corresponds to perfect performance (of a random classifier). The other control metrics are calculated using the two halves of the real test dataset.

Simulator	Cosine distance	Euclidean distance	MMD	RF AUROC	miLISI
SERGIO (Clean)	0.96998	5331	9.052	1.00	1.00
SERGIO (Synth)	0.86783	4913	6.247	1.00	1.00
GRouNdGAN	0.00057	182	0.026	0.54	1.89
Control	0.00019	99	0.012	0.50	1.91

Supplementary Table 5: Training set performance of SERGIO in generating realistic scRNA-seq data using the PBMC-CTL dataset. SERGIO (Clean) and SERGIO (Synth) refer to the simulated data before and after distribution matching through the addition of technical noise, respectively. The metrics are calculated between a simulated dataset of 1000 cells and 1000 real cells randomly sampled from the training set. For both SERGIO and GRouNdGAN, the same GRN was imposed. In this GRN, each gene was regulated by 15 TFs (constructed using GRNBoost2 from the experimental training set). For the first three metrics, a value closer to zero is preferred, for RF AUROC a value closer to 0.5 is preferred, and for miLISI a value closer to 2 is preferred. For the first two metrics, the values correspond to the distance of the mean centroids of the real and simulated cells. The RF AUROC of control corresponds to perfect performance (of a random classifier). The other control metrics are calculated using the two halves of the real training dataset.

Simulator	Cosine distance	Euclidean distance	MMD	RF AUROC	miLISI
SERGIO (Clean)	0.96867	5248	9.026	1.00	1.00
SERGIO (Synth)	0.86998	5000	6.215	1.00	1.00
GRouNdGAN	0.00018	95	0.014	0.53	1.90
Control	0.00013	86	0.012	0.50	1.91

Supplementary Table 6: Cell types present in the BoneMarrow dataset, their abbreviations, and the list of marker genes associated with each used to annotate nodes of the PAGA graphs. The markers genes were obtained from the link (https://scanpy-tutorials.readthedocs.io/en/latest/paga-paul15.html) [9]. Marker genes not included in the top 1000 highly variable genes were excluded from this table and not used in our analyses.

**Cell type**	**Abbreviation**	**Marker gene(s)**
Erythroid cells	Ery	*Gata1, Klf1, Hba-a2*
Neutrophils	Neu	*Elane, Cebpe, Ctsg, Mpo*
Monocytes	Mo	*Irf8, Csf1r, Ctsg, Mpo*
Megakaryocytes	Mk	*Itga2b* (encodes protein CD41), *Pbx1, Sdpr, Vwf*
Basophils	Baso	*Mcpt8, Prss34*
Mast cells	Ma	*Cma1, Gzmb*
Mast cells & Basophils	Ma_Baso	*Cpa3*

Supplementary Table 7: The effect of cell type specific TF knockout on the miLISI values of each cell type in the PBMC-All dataset. The first column shows the cell type, the second column shows the percentage of that cell type in the data, the third and fourth columns show miLISI before and after knockout (KO) of top three TFs (shown in the seventh column), respectively. The fifth and sixth columns show the miLISI of other cell types before and after the KO experiment.

**Cell type**	**Perc.**	**miLISI before KO**	**miLISI after KO**	**miLISI of other cells before KO**	**miLISI of other cells after KO**	**Top DE TFs**
CD14+ Monocytes	4.75	1.97	1.42	1.94	1.91	*SPI1, CEBPD, JUND*
CD19+ B cells	8.7	1.96	1.42	1.94	1.92	*SPIB, IRF8, LYL1*
CD56+ Natural Killer (NK) cells	11.8	1.93	1.71	1.95	1.92	*HOPX, ASCL2, ZNF683*
Dendritic cells	3.4	1.98	1.46	1.94	1.89	*SPI1, CEBPD, LYL1*

Supplementary Table 8: The effect of cell type specific TF knockout on the miLISI values of each cell type in the Tumor-All dataset. The first column shows the cell type, the second column shows the percentage of that cell type in the data, the third and fourth columns show miLISI before and after knockout (KO) of top three TFs (shown in the seventh column), respectively. The fifth and sixth columns show the miLISI of other cell types before and after the KO experiment.

**Cell type**	**Perc.**	**miLISI before KO**	**miLISI after KO**	**miLISI of other cells before KO**	**miLISI of other cells after KO**	**Top DE TFs**
Malignant cells	65.04	1.93	1.76	1.93	1.85	*ATF5, TSC22D3, ZNF860*
T cells	25.26	1.92	1.27	1.93	1.62	*TCF7, BATF, MAF*
B cells	11.8	1.93	1.57	1.93	1.75	*ID3, KLF2, JUN*
Plasma cells	0.26	1.91	1.18	1.93	1.76	*XBP1, PRDM1, JUN*

Supplementary Figures

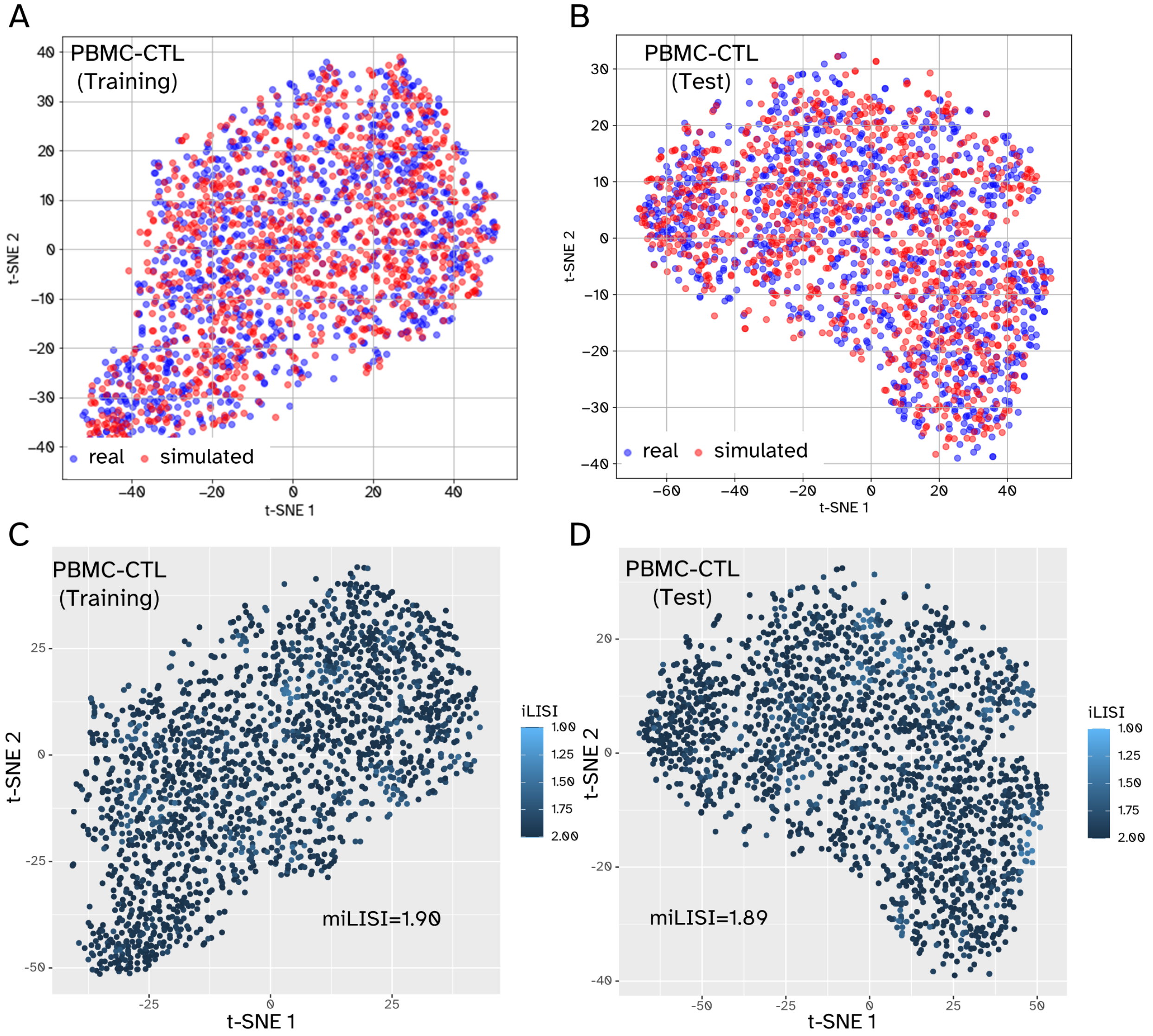

Supplementary Fig. 1: Real (experimental) and GRouNdGAN-simulated scRNA-seq data based on the PBMC-CTL dataset. All plots correspond to 1000 simulated cells and 1000 real cells. Each gene in the GRN of GRouNdGAN is regulated by 15 TFs (identified using GRNBoost2 from the experimental training dataset). Panels A and B show t-SNE plots of simulated cells (red) and real cells (blue). Panels C and D show the iLISI values of each datapoint and the average iLISI score of the data (miLISI). Panels A and C correspond to comparison between simulated cells and a random set of real cells in the training set, while panels B and D correspond to comparison between simulated cells and all the real cells in the test set. Source data are provided as a Source Data file.

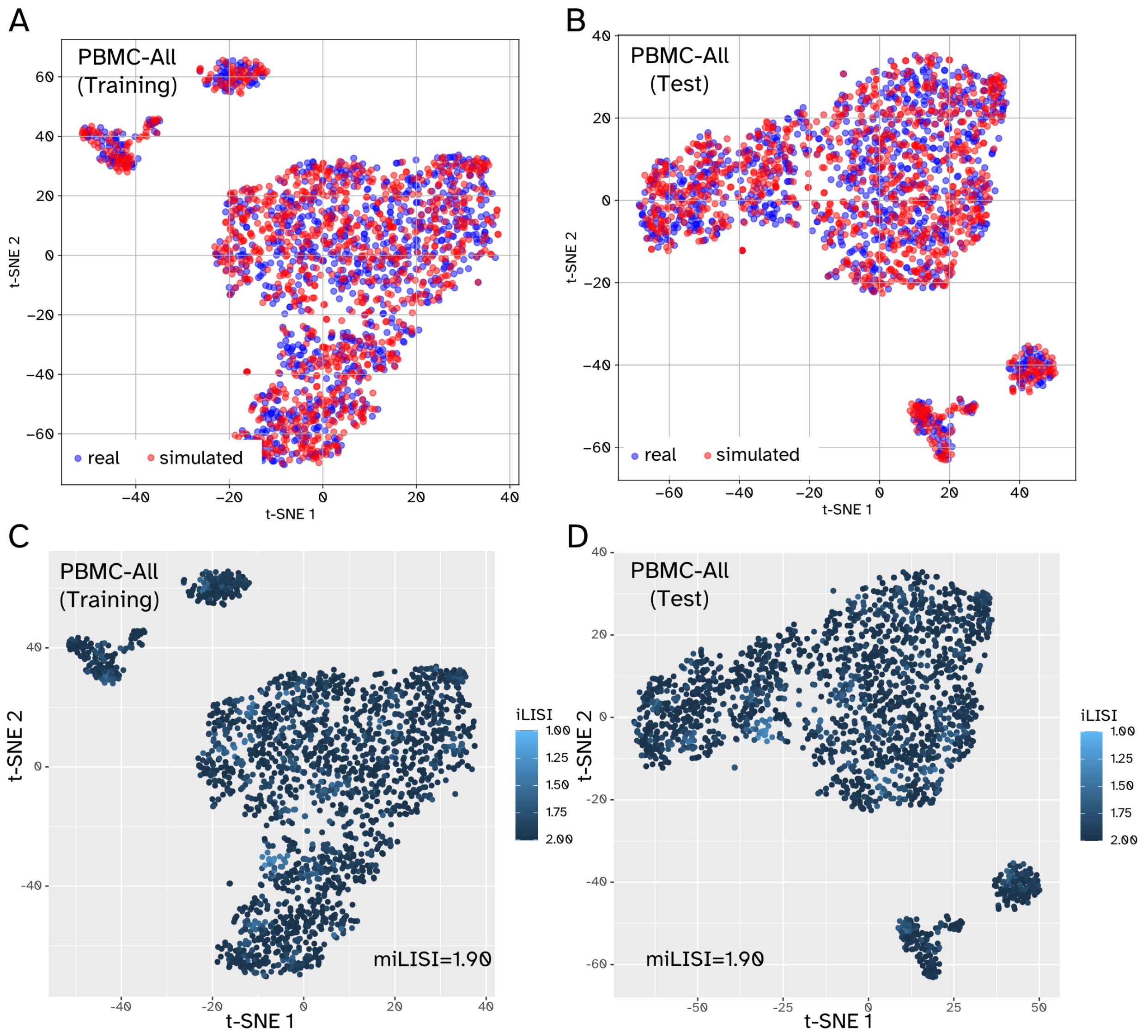

Supplementary Fig. 2: Real (experimental) and GRouNdGAN-simulated scRNA-seq data using the PBMC-All dataset. All plots correspond to 1000 simulated cells and 1000 real cells. Each gene in the GRN of GRouNdGAN is regulated by 15 TFs (identified using GRNBoost2 from the experimental training dataset). Panels A and B show t-SNE plots of simulated cells (red) and real cells (blue). Panels C and D show the iLISI values of each datapoint and the average iLISI score of the data (miLISI). Panels A and C correspond to comparison between simulated cells and a random set of real cells in the training set, while panels B and D correspond to comparison between simulated cells and all the real cells in the test set. Source data are provided as a Source Data file.

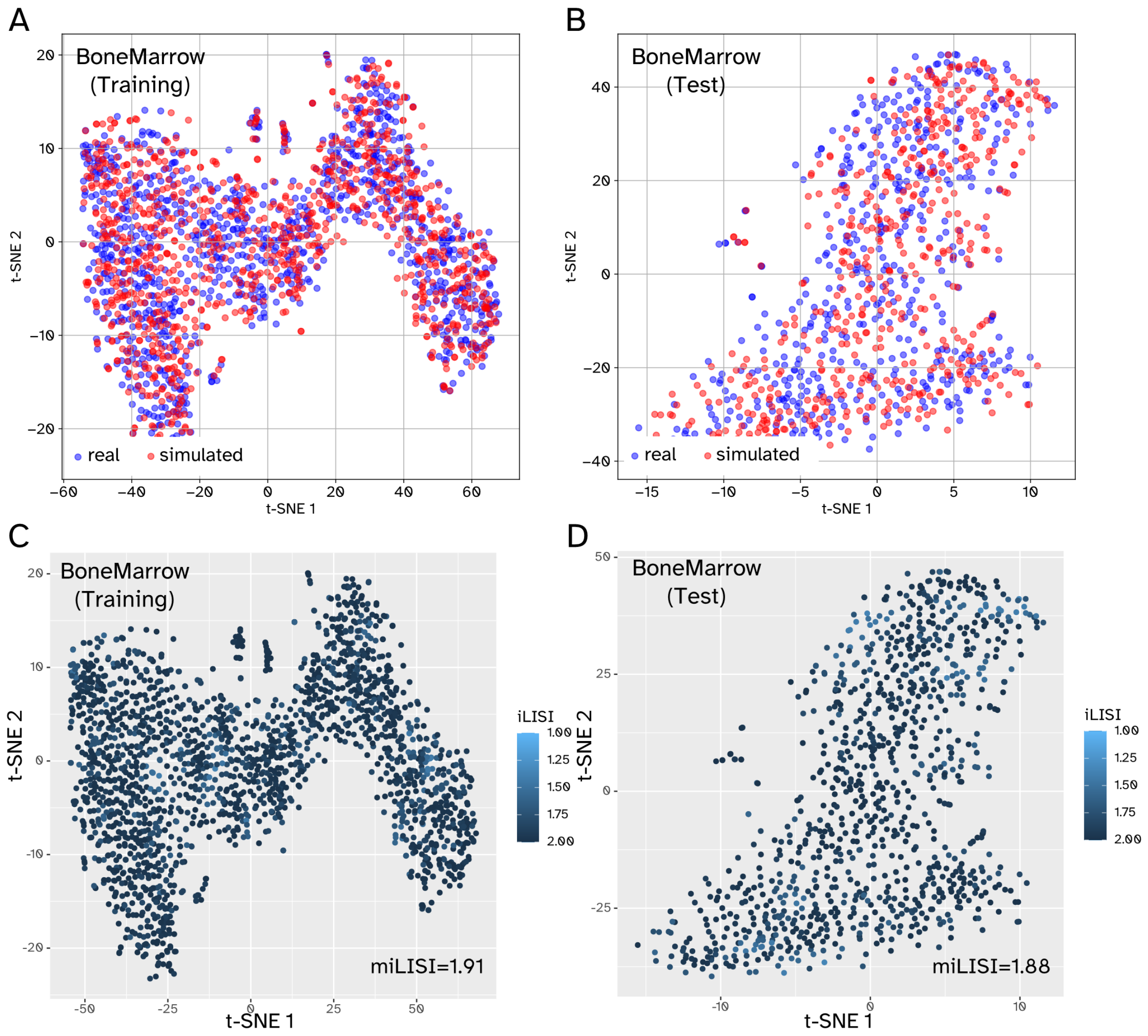

Supplementary Fig. 3: Real experimental and GRouNdGAN-simulated scRNA-seq data using the BoneMarrow dataset. All plots correspond to 500 simulated cells and 500 real cells. Each gene in the GRN of GRouNdGAN is regulated by 15 TFs (identified using GRNBoost2 from the experimental training dataset). Panels A and B show t-SNE plots of simulated cells (red) and real cells (blue). Panels C and D show the iLISI values of each datapoint and the average iLISI score of the data (miLISI). Panels A and C correspond to comparison between simulated cells and a random set of real cells in the training set, while panels B and D correspond to comparison between simulated cells and all the real cells in the test set. Source data are provided as a Source Data file.

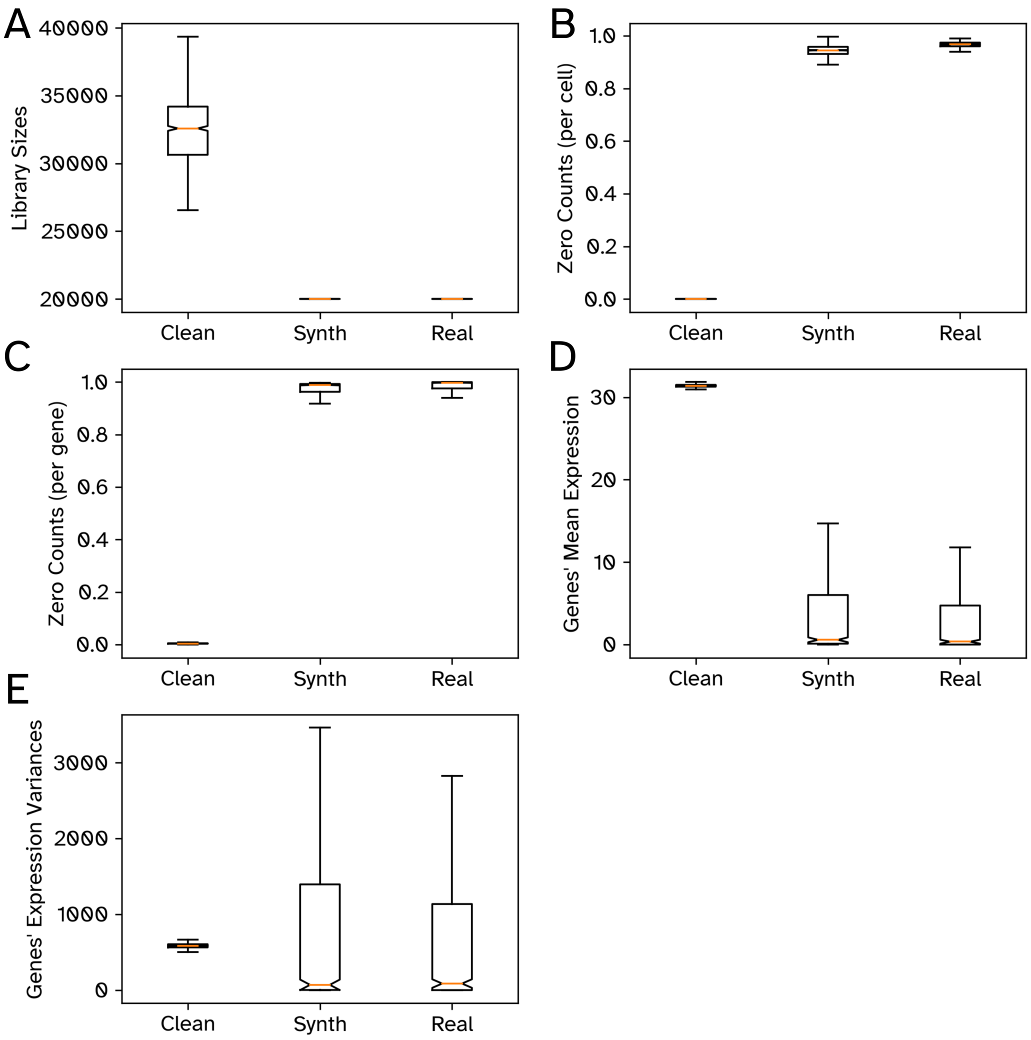

Supplementary Fig. 4: SERGIO's statistical parameters used to fine-tune its output to the PBMC-CTL reference dataset (related to Supplementary Tables 4 and 5). "Clean" corresponds to the output of SERGIO before addition of technical noise, "Synth" refers to the dataset when technical noise was added to best match the reference dataset, and "Real" refers to the experimental PBMC-CTL reference dataset. The imposed GRN was the same GRN used with GRouNdGAN in Table 1. Panel A compares the library size distributions. Panels B and C show the distribution of zero counts per cell (normalized by number of genes) and per gene (normalized by number of cells), respectively. Panels D and E show the distribution of genes' expression means and variances, respectively. Source data are provided as a Source Data file.

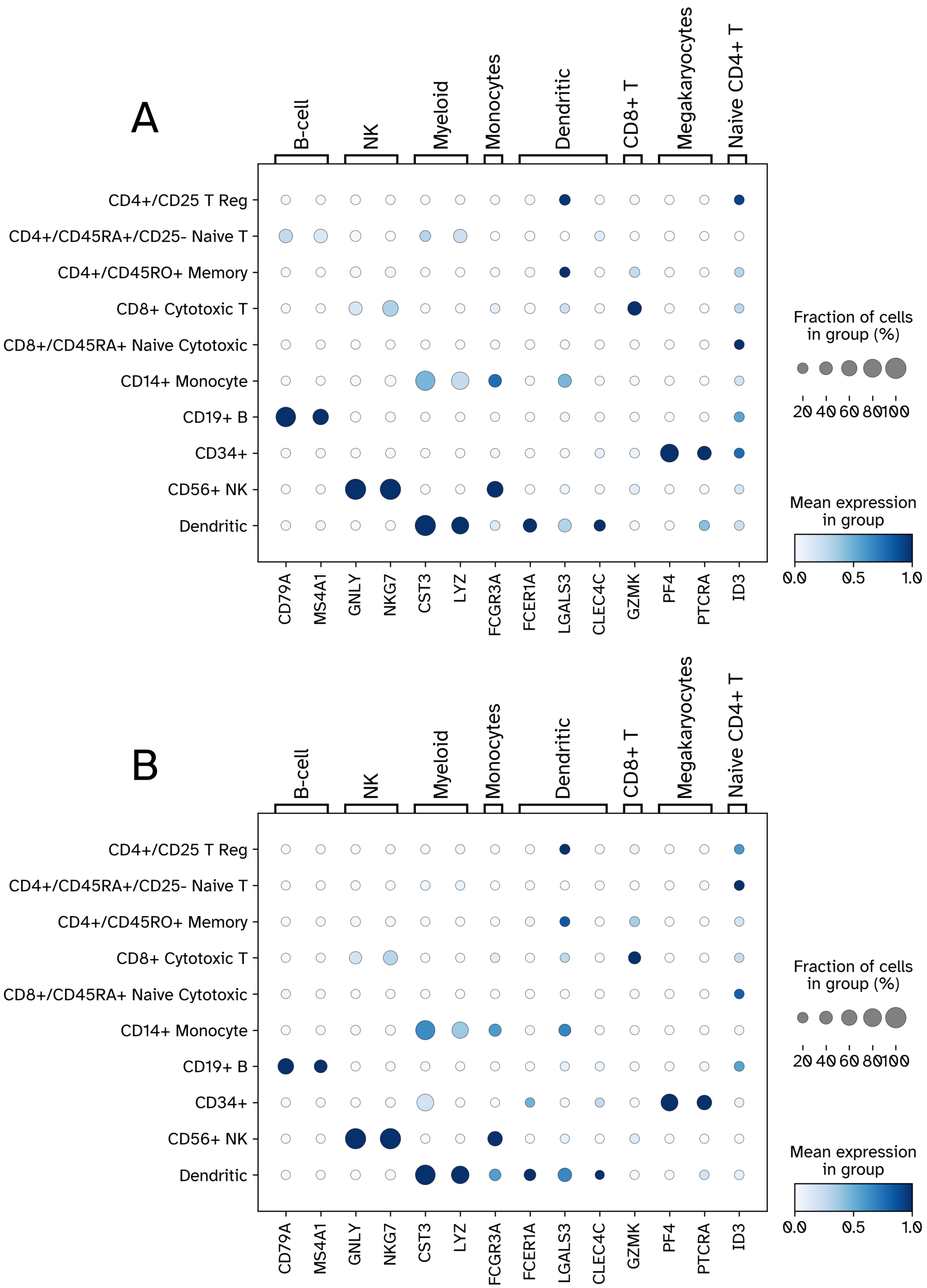

Supplementary Fig. 5: Cell type markers in the experimental and GRouNdGAN-generated data. The dot plots of panels A (simulated data) and B (experimental data) visualize the fraction of cells per cell type expressing a marker through dot size and the mean standardized marker expression through color intensity. Source data are provided as a Source Data file.

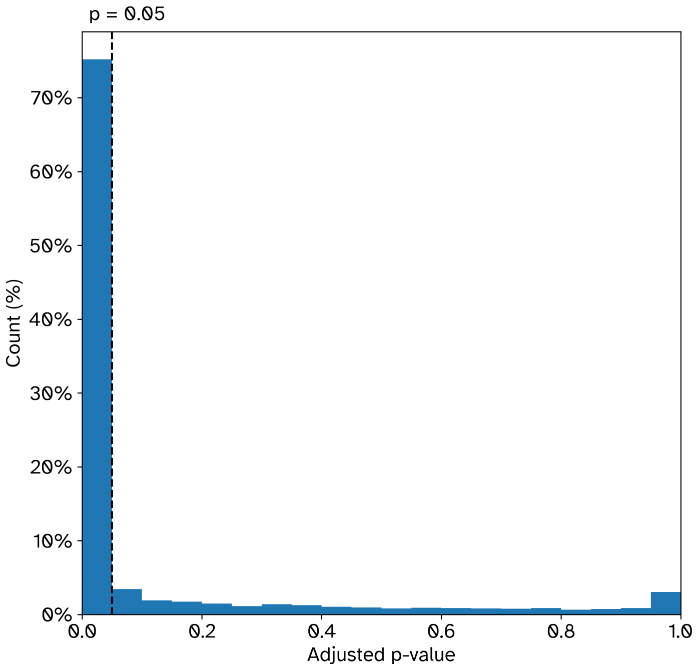

Supplementary Fig. 6: The distribution of adjusted p-values corresponding to the TF perturbation study on the PBMC-CTL dataset (n = 1000 cells), when the GRN was found using PPCOR. The p-values are obtained using two-sided Wilcoxon signed ranked tests and are adjusted for multiple hypotheses following the Benjamini-Hochberg procedure. Each gene in the imposed GRN of GRouNdGAN is regulated by 15 TFs (identified using PPCOR from the experimental training set). Source data are provided as a Source Data file.

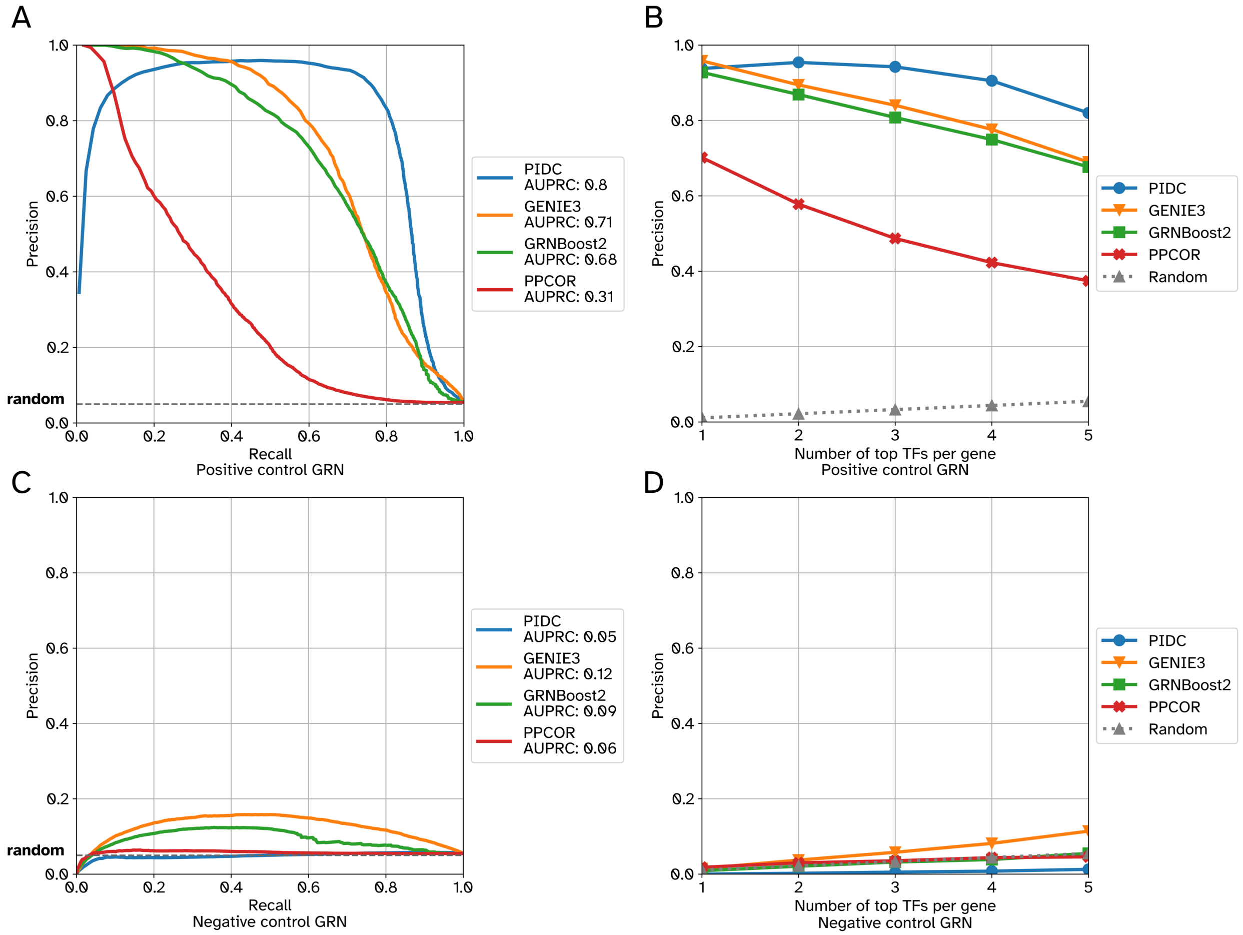

Supplementary Fig. 7: Performance of different GRN inference algorithms in recovering the imposed edges versus unimposed edges using data generated by GRouNdGAN based on the PBMC-CTL dataset (n = 19,773 simulated cells). Top row shows the AUPRC (A) and Precision at k (per gene) (B) when the imposed edges (positive control GRN) were considered the ground truth. Bottom row shows the AUPRC (C) and Precision at k (per gene) (D) when the unimposed edges (negative control GRN) were considered the ground truth. Precision at k (per gene) refers to the precision when top k TFs for each gene is used to form the reconstructed GRN. Source data are provided as a Source Data file.

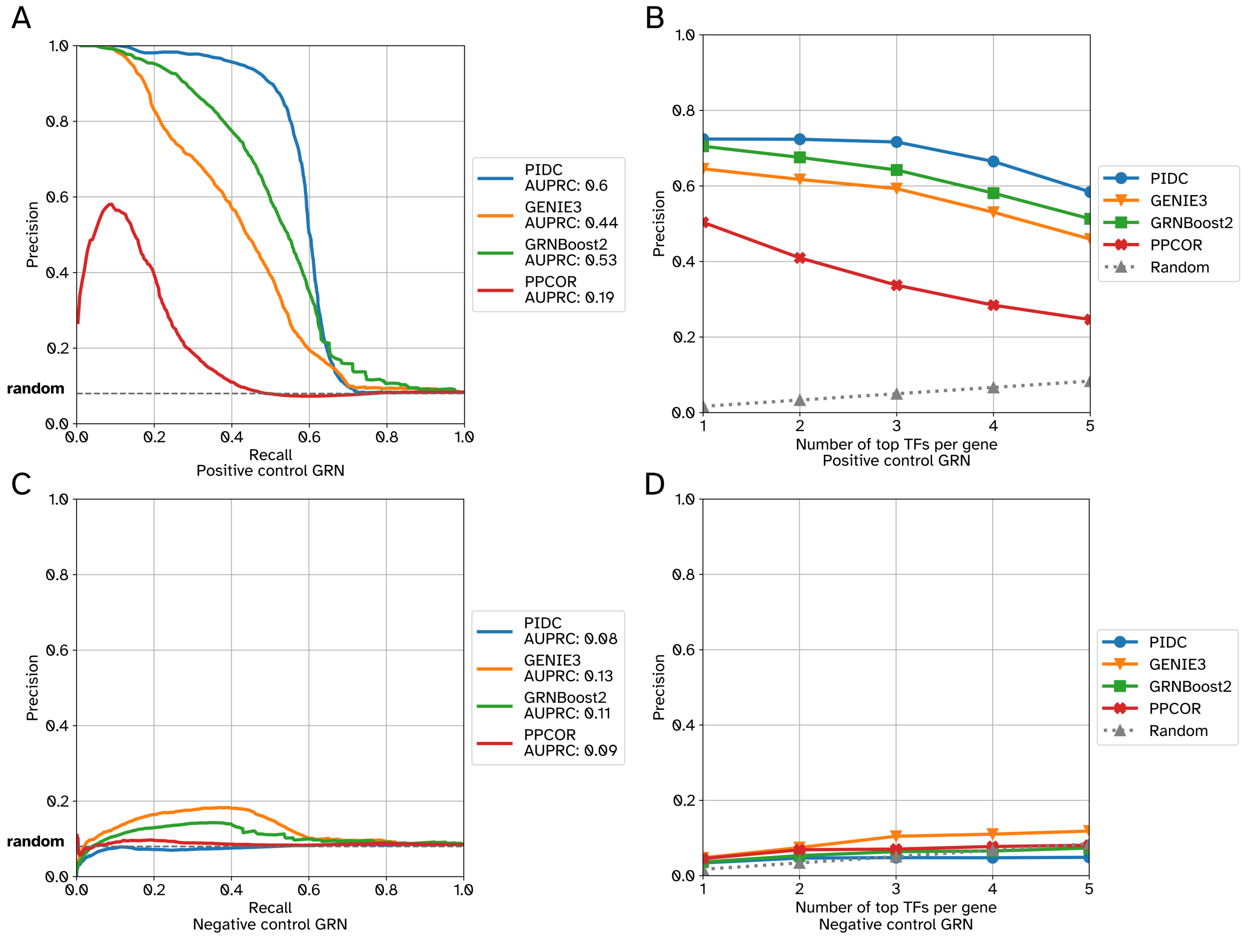

Supplementary Fig. 8: Performance of different GRN inference algorithms in recovering the imposed edges versus unimposed edges using data generated by GRouNdGAN based on the PBMC-All dataset (n = 67,579 simulated cells). Top row shows the AUPRC (A) and Precision at k (per gene) (B) when the imposed edges (positive control GRN) were considered the ground truth. Bottom row shows the AUPRC (C) and Precision at k (per gene) (D) when the unimposed edges (negative control GRN) were considered the ground truth. Precision at k (per gene) refers to the precision when top k TFs for each gene is used to form the reconstructed GRN. Source data are provided as a Source Data file.

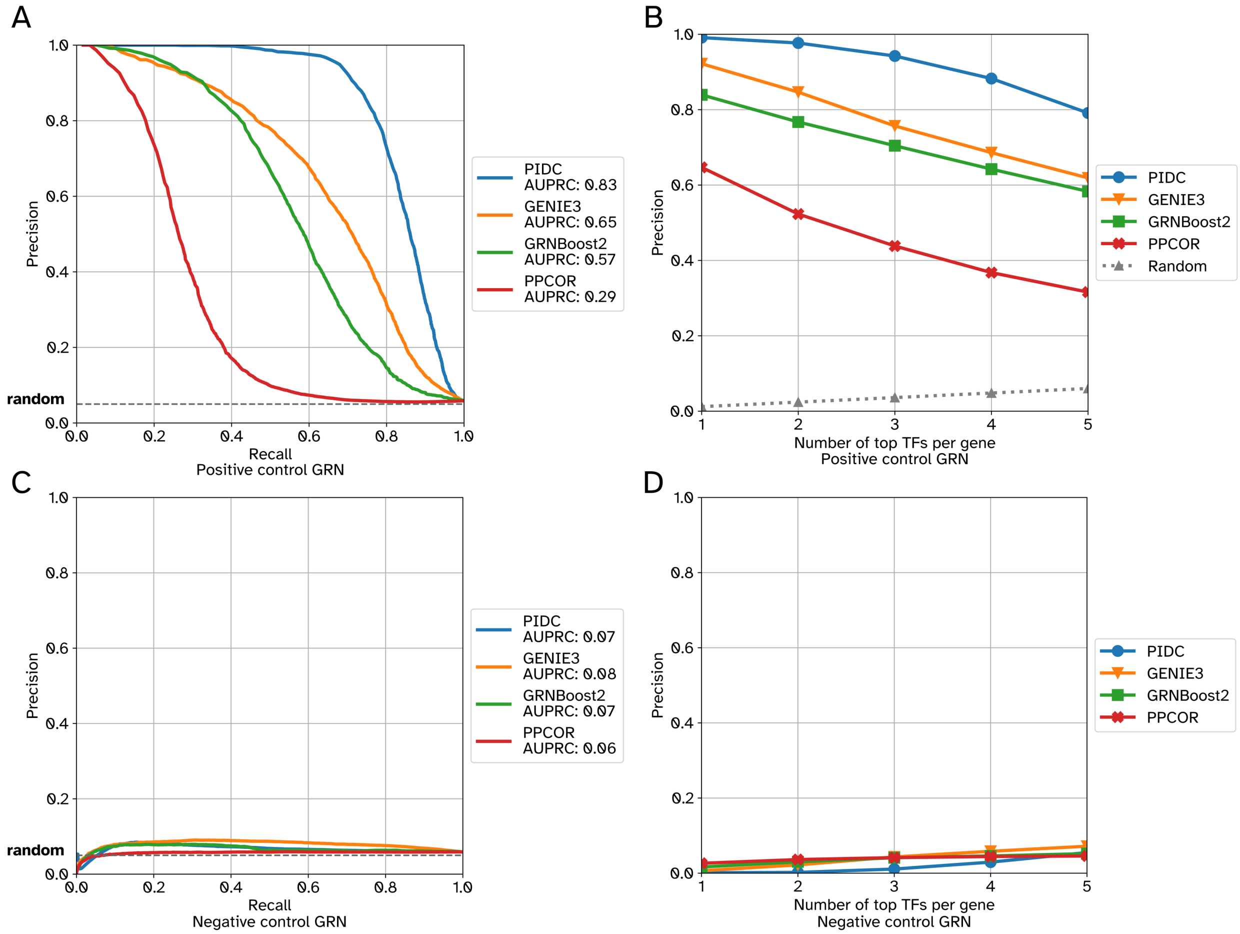

Supplementary Fig. 9: Performance of different GRN inference algorithms in recovering the imposed edges versus unimposed edges using data generated by GRouNdGAN based on the BoneMarrow dataset (n = 2,230 simulated cells). Top row shows the AUPRC (A) and Precision at k (per gene) (B) when the imposed edges (positive control GRN) were considered the ground truth. Bottom row shows the AUPRC (C) and Precision at k (per gene) (D) when the unimposed edges (negative control GRN) were considered the ground truth. Precision at k (per gene) refers to the precision when top k TFs for each gene is used to form the reconstructed GRN. Source data are provided as a Source Data file.

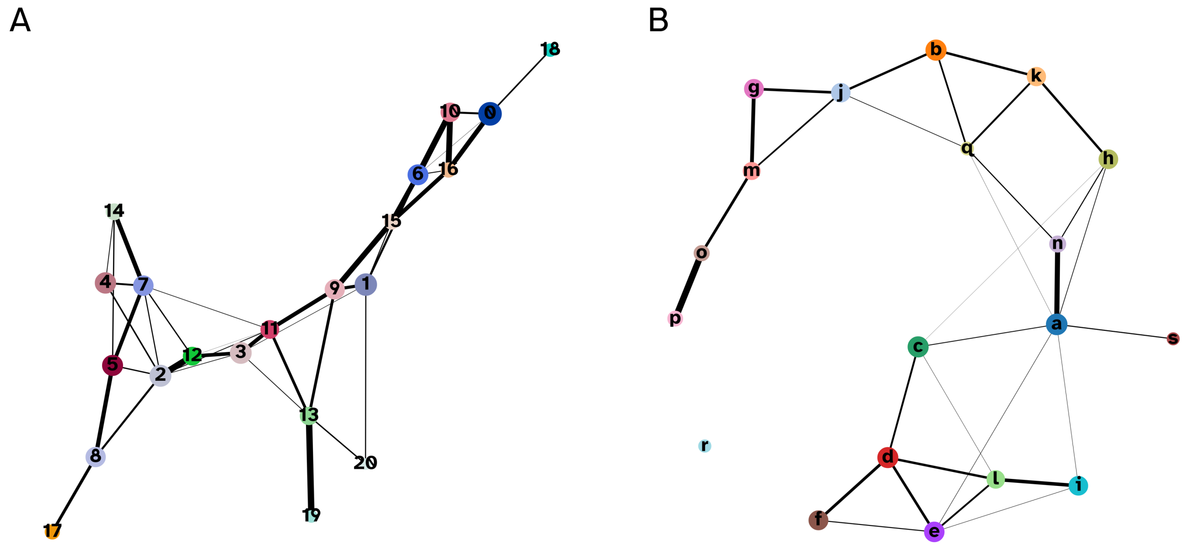

Supplementary Fig. 10: PAGA generated graphs for the BoneMarrow dataset. Nodes represent Louvain clusters capturing discrete states, edges show transitions among these states, and edge weights show-case the confidence in the existence of connections. A) The PAGA graph computed from GRouNdGAN-generated data comprising of 21 clusters. Each gene in the GRN of GRouNdGAN is regulated by 15 TFs (identified using GRNBoost2 from the real dataset) and the same number of cells as the original dataset was generated. B) The PAGA graph computed from the original BoneMarrow dataset comprising of 19 clusters. Low-connectivity edges below a threshold of 0.01 were discarded. Source data are provided as a Source Data file.

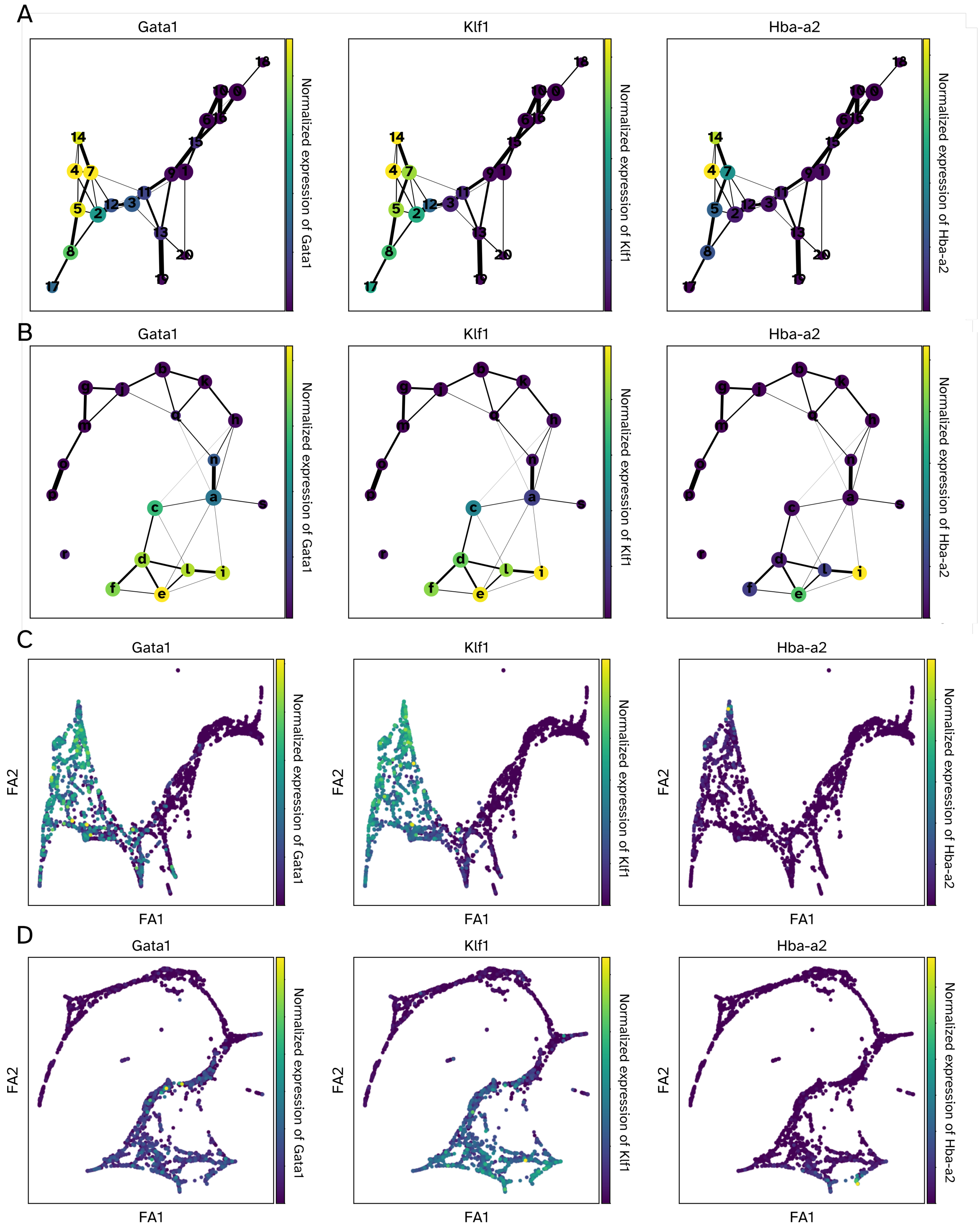

Supplementary Fig. 11: Erythroid cells' marker genes activation patterns in experimental and simulated data. Panels A and C show the normalized gene expression of the marker genes in PAGA graphs for the simulated and the experimental data, respectively. Nodes correspond to Louvain clusters capturing discrete states and edges show transitions among these states. Panels B and D show PAGA-initialized single-cell embeddings obtained using ForceAtlas2. In all figures,

colors yellow and purple show the highest and lowest normalized gene expression value, respectively. Source data are provided as a Source Data file.

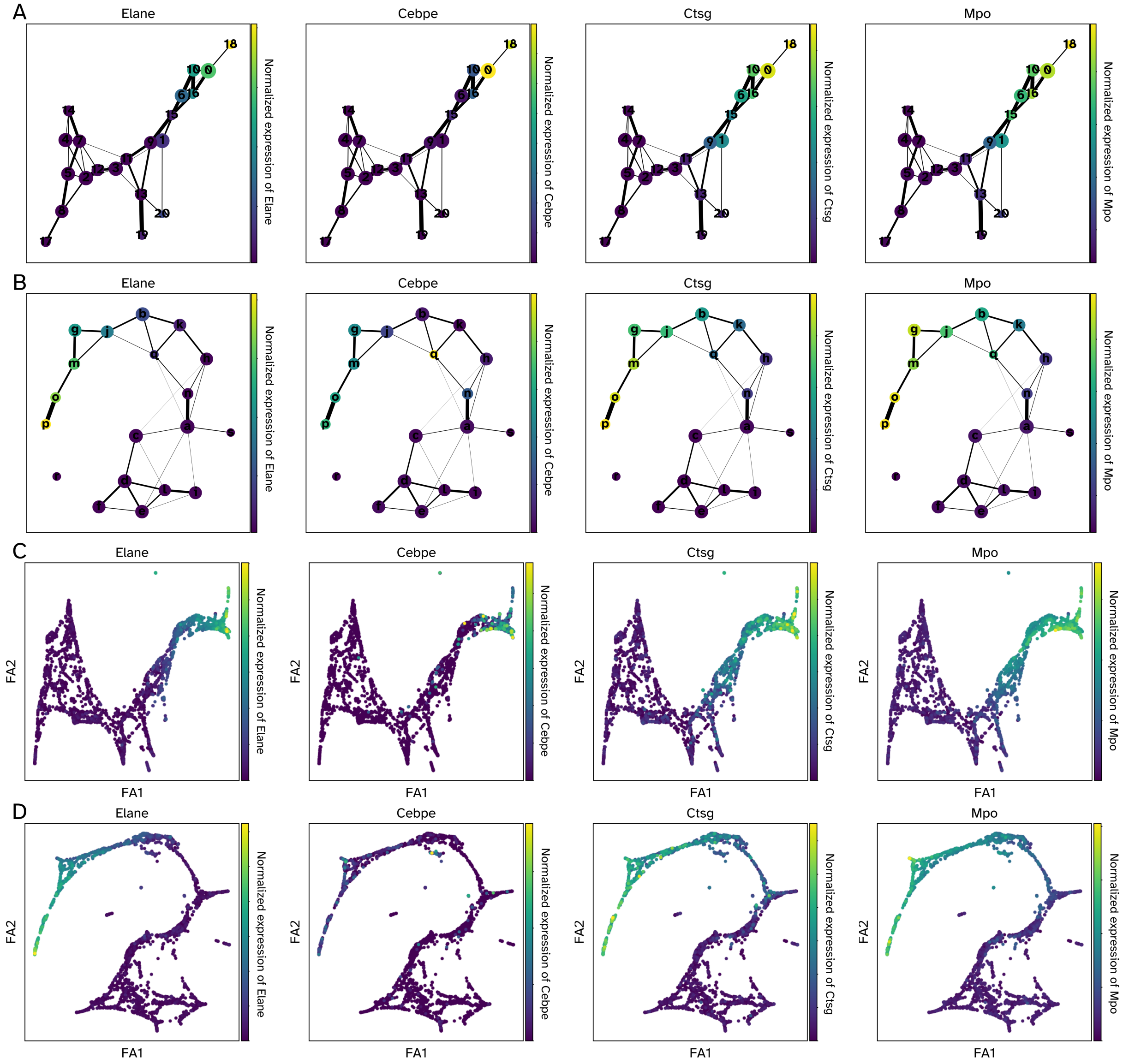

Supplementary Fig. 12: Neutrophils' marker genes activation patterns in experimental and simulated data. Panels A and C show the normalized gene expression of the marker genes in PAGA graphs for the simulated and the experimental data, respectively. Nodes correspond to Louvain clusters capturing discrete states and edges show transitions among these states. Panels B and D show PAGA-initialized single-cell embeddings obtained using ForceAtlas2. In all figures, colors yellow and purple show the highest and lowest normalized gene expression value, respectively. Source data are provided as a Source Data file.

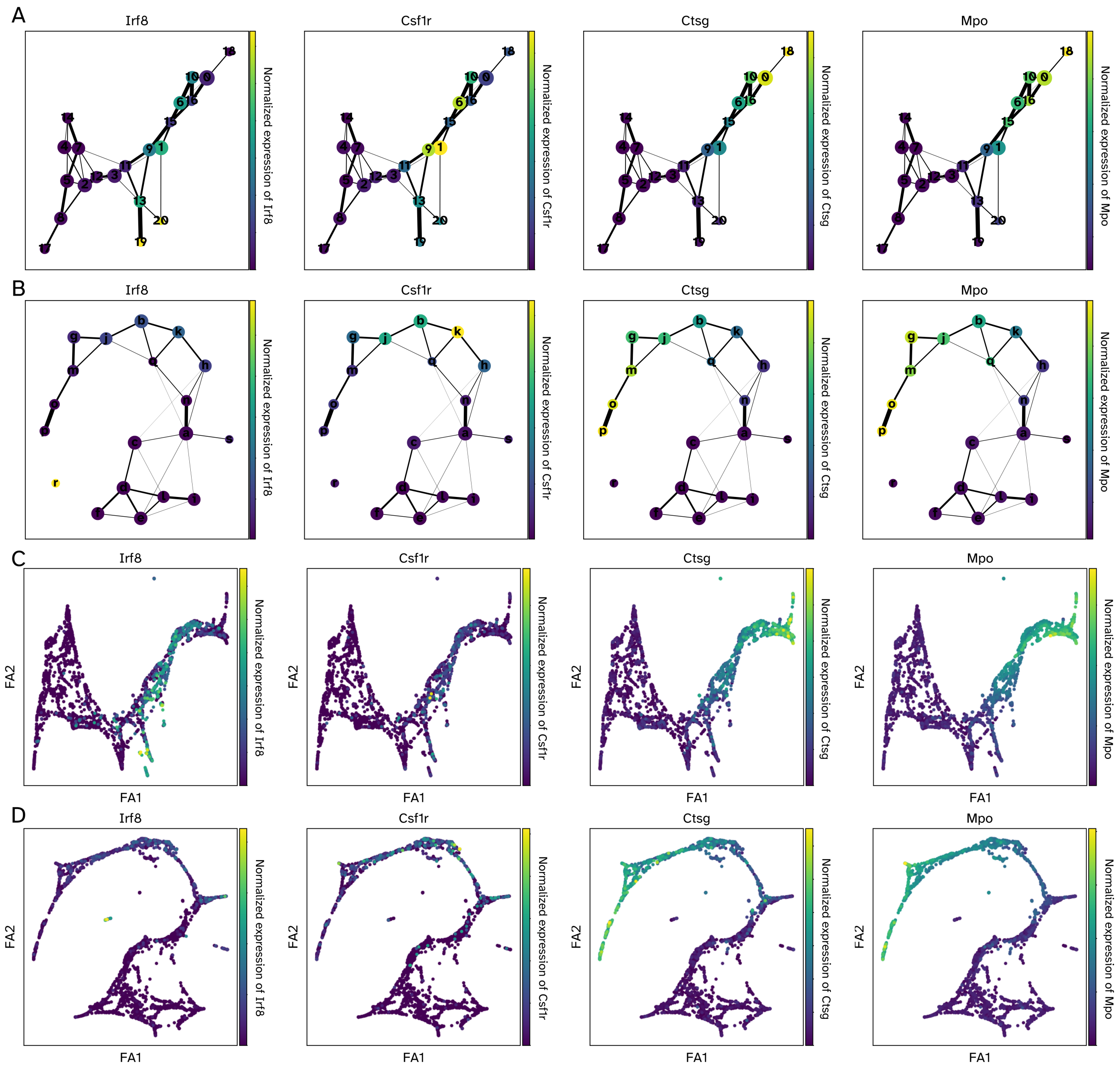

Supplementary Fig. 13: Monocytes' marker genes activation patterns in experimental and simulated data. Panels A and C show the normalized gene expression of the marker genes in PAGA graphs for the simulated and the experimental data, respectively. Nodes correspond to Louvain clusters capturing discrete states and edges show transitions among these states. Panels B and D show PAGA-initialized single-cell embeddings obtained using ForceAtlas2. In all figures, colors yellow and purple show the highest and lowest normalized gene expression value, respectively. Source data are provided as a Source Data file.

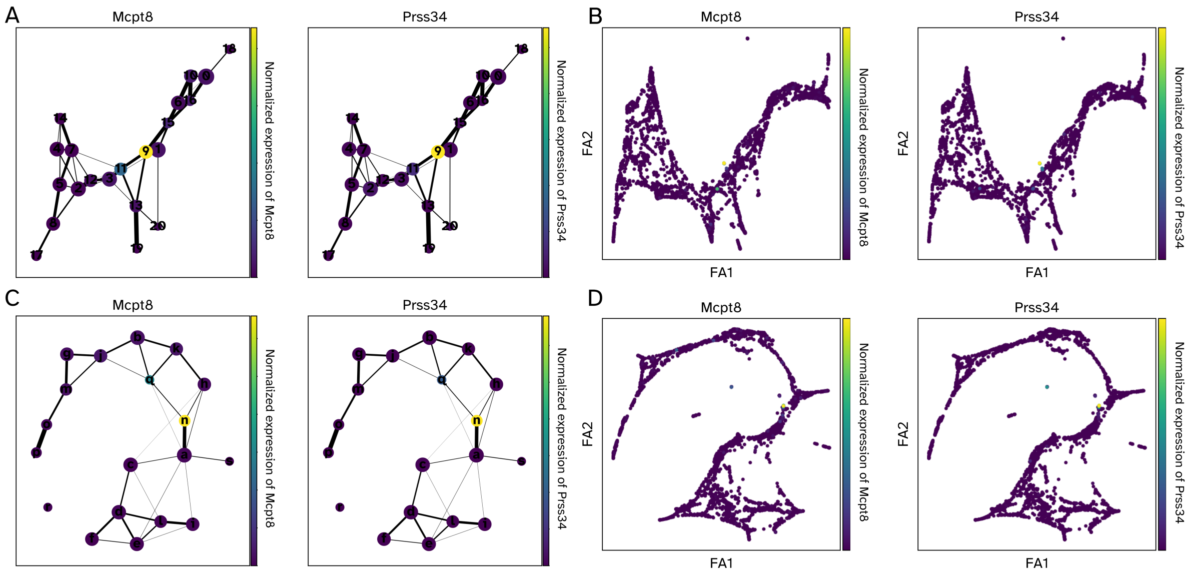

Supplementary Fig. 14: Basophils' marker genes activation patterns in experimental and simulated data. Panels A and C show the normalized gene expression of the marker genes in PAGA graphs for the simulated and the experimental data, respectively. Nodes correspond to Louvain clusters capturing discrete states and edges show transitions among these states. Panels B and D show PAGA-initialized single-cell embeddings obtained using ForceAtlas2. In all figures, colors yellow and purple show the highest and lowest normalized gene expression value, respectively. Source data are provided as a Source Data file.

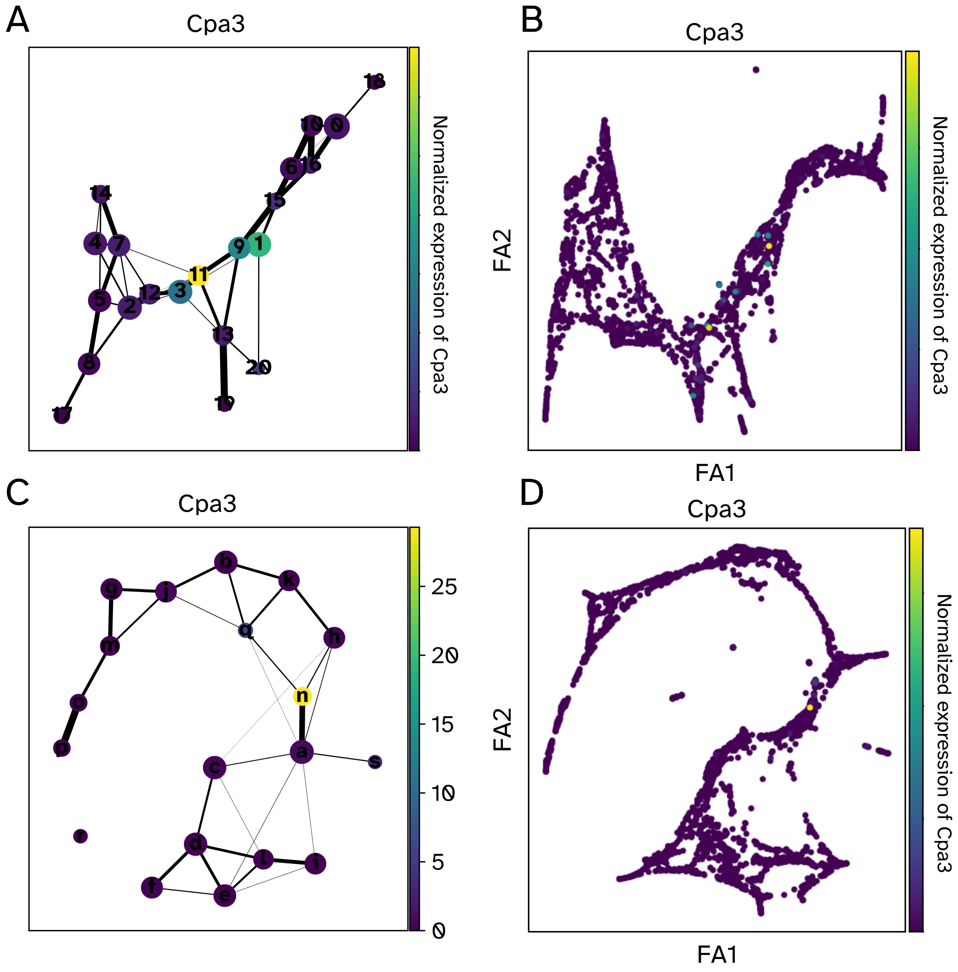

Supplementary Fig. 15: Basophils and Mast cells' marker gene activation patterns in experimental and simulated data. Panels A and C show the normalized gene expression of the marker gene in PAGA graphs for the simulated and the experimental data, respectively. Nodes correspond to Louvain clusters capturing discrete states and edges show transitions among these states. Panels B and D show PAGA-initialized single-cell embeddings obtained using ForceAtlas2. In all figures, colors yellow and purple show the highest and lowest normalized gene expression value, respectively. Source data are provided as a Source Data file.

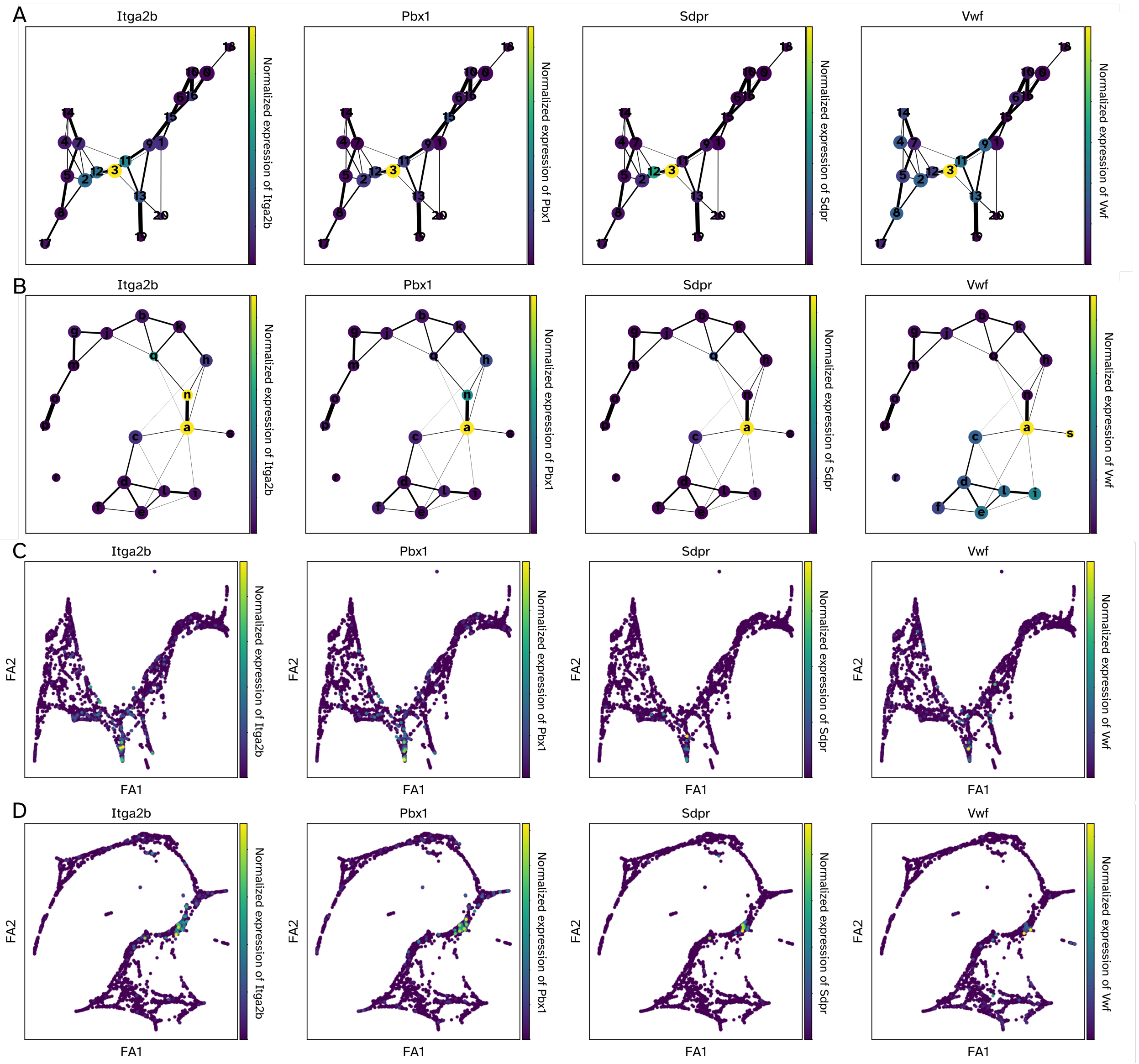

Supplementary Fig. 16: Megakaryocytes' marker genes activation patterns in experimental and simulated data. Panels A and C show the normalized gene expression of the marker genes in PAGA graphs for the simulated and the experimental data, respectively. Nodes correspond to Louvain clusters capturing discrete states and edges show transitions among these states. Panels B and D show PAGA-initialized single-cell embeddings obtained using ForceAtlas2. In all figures, colors yellow and purple show the highest and lowest normalized gene expression value, respectively. Source data are provided as a Source Data file.

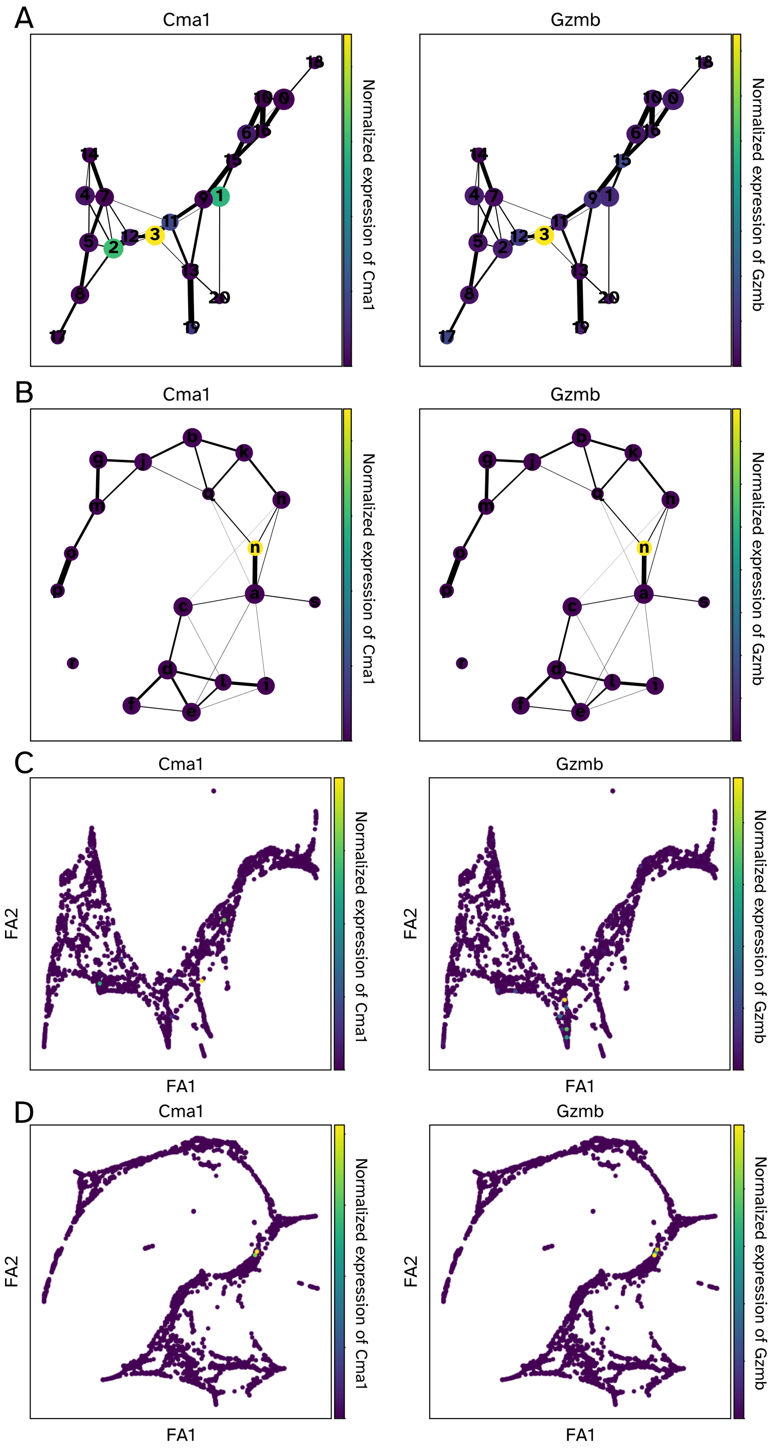

Supplementary Fig. 17: Mast cells' marker genes activation patterns in experimental and simulated data. Panels A and C show the normalized gene expression of the marker genes in PAGA graphs for the simulated and the experimental data, respectively. Nodes correspond to Louvain clusters capturing discrete states and edges show transitions among these states. Panels B and D show PAGA-initialized single-cell embeddings obtained using ForceAtlas2. In all figures,

colors yellow and purple show the highest and lowest normalized gene expression value, respectively. Source data are provided as a Source Data file.

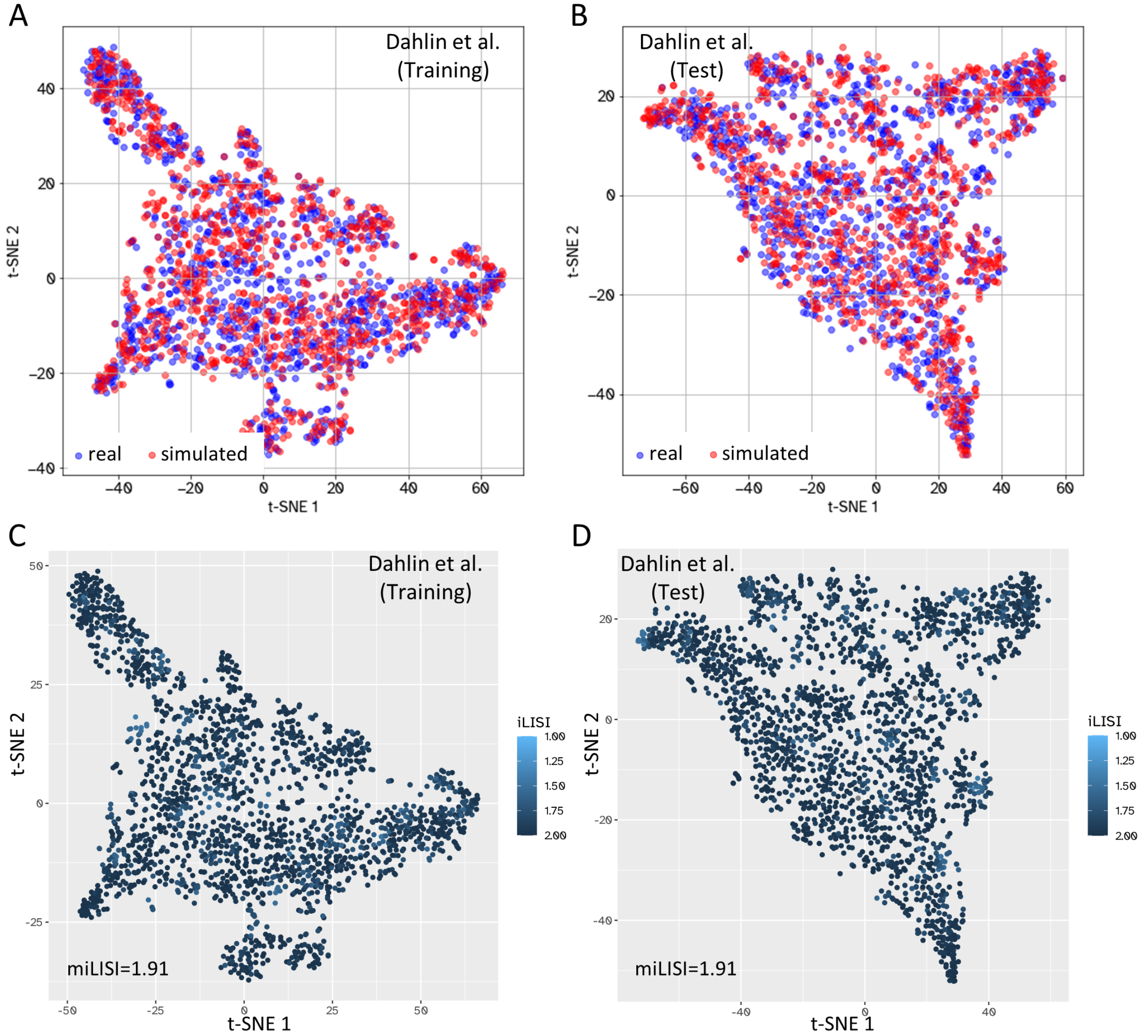

Supplementary Fig. 18: Real (experimental) and GRouNdGAN-simulated scRNA-seq data based on the Dahlin dataset. All plots correspond to 1000 simulated cells and 1000 real cells. Each gene in the GRN of GRouNdGAN is regulated by 15 TFs (identified using GRNBoost2 from the experimental training dataset). Panels A and B show t-SNE plots of simulated cells (red) and real cells (blue). Panels C and D show the iLISI values of each datapoint and the average iLISI score of the data (miLISI). Panels A and C correspond to comparison between simulated cells and a random set of real cells in the training set, while panels B and D correspond to comparison between simulated cells and all the real cells in the test set. Source data are provided as a Source Data file.

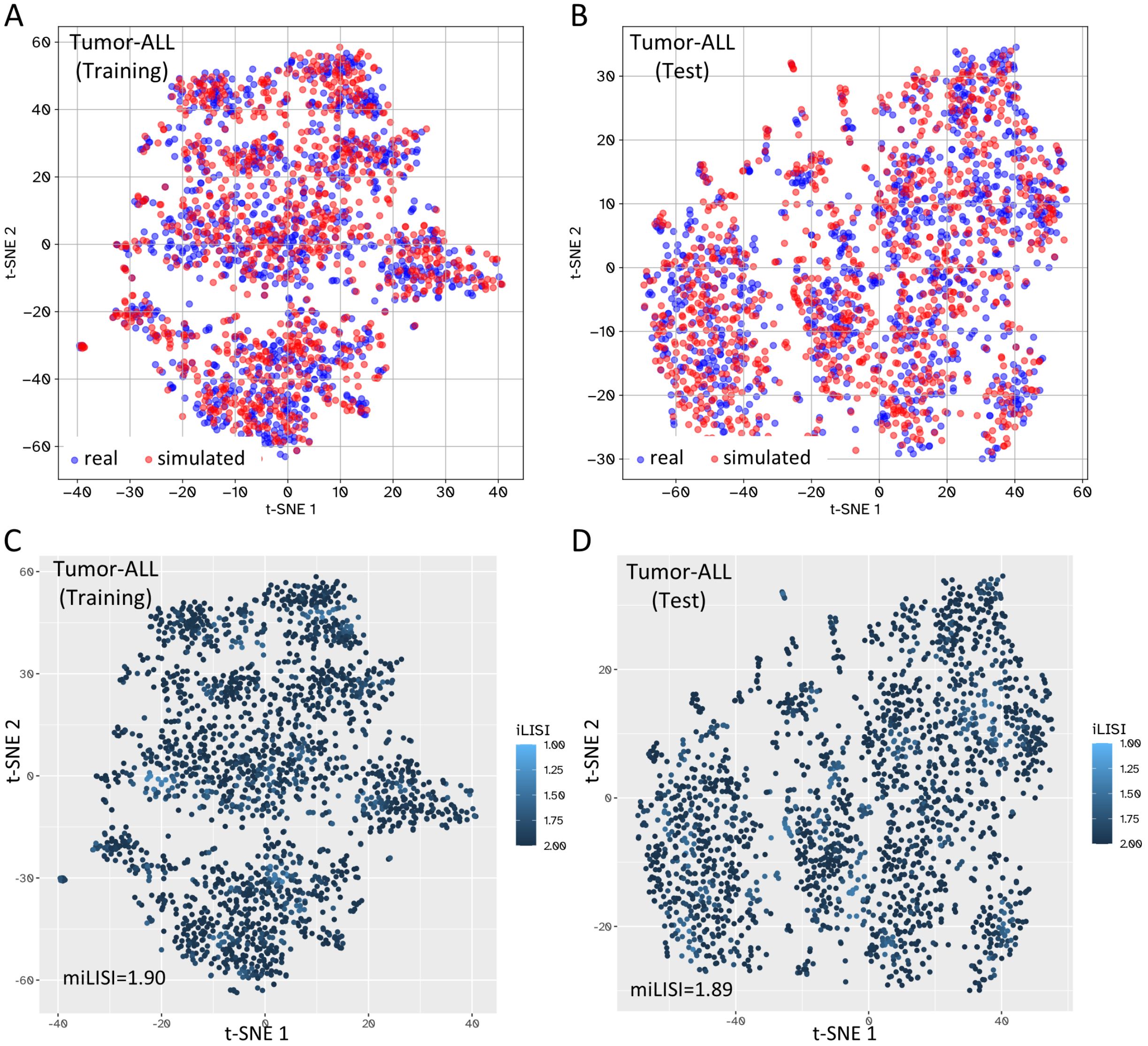

Supplementary Fig. 19: Real (experimental) and GRouNdGAN-simulated scRNA-seq data based on the Tumor-All dataset. All plots correspond to 1000 simulated cells and 1000 real cells. Each gene in the GRN of GRouNdGAN is regulated by 15 TFs (identified using GRNBoost2 from the experimental training dataset). Panels A and B show t-SNE plots of simulated cells (red) and real cells (blue). Panels C and D show the iLISI values of each datapoint and the average iLISI score of the data (miLISI). Panels A and C correspond to comparison between simulated cells and a random set of real cells in the training set, while panels B and D correspond to comparison between simulated cells and all the real cells in the test set. Source data are provided as a Source Data file.

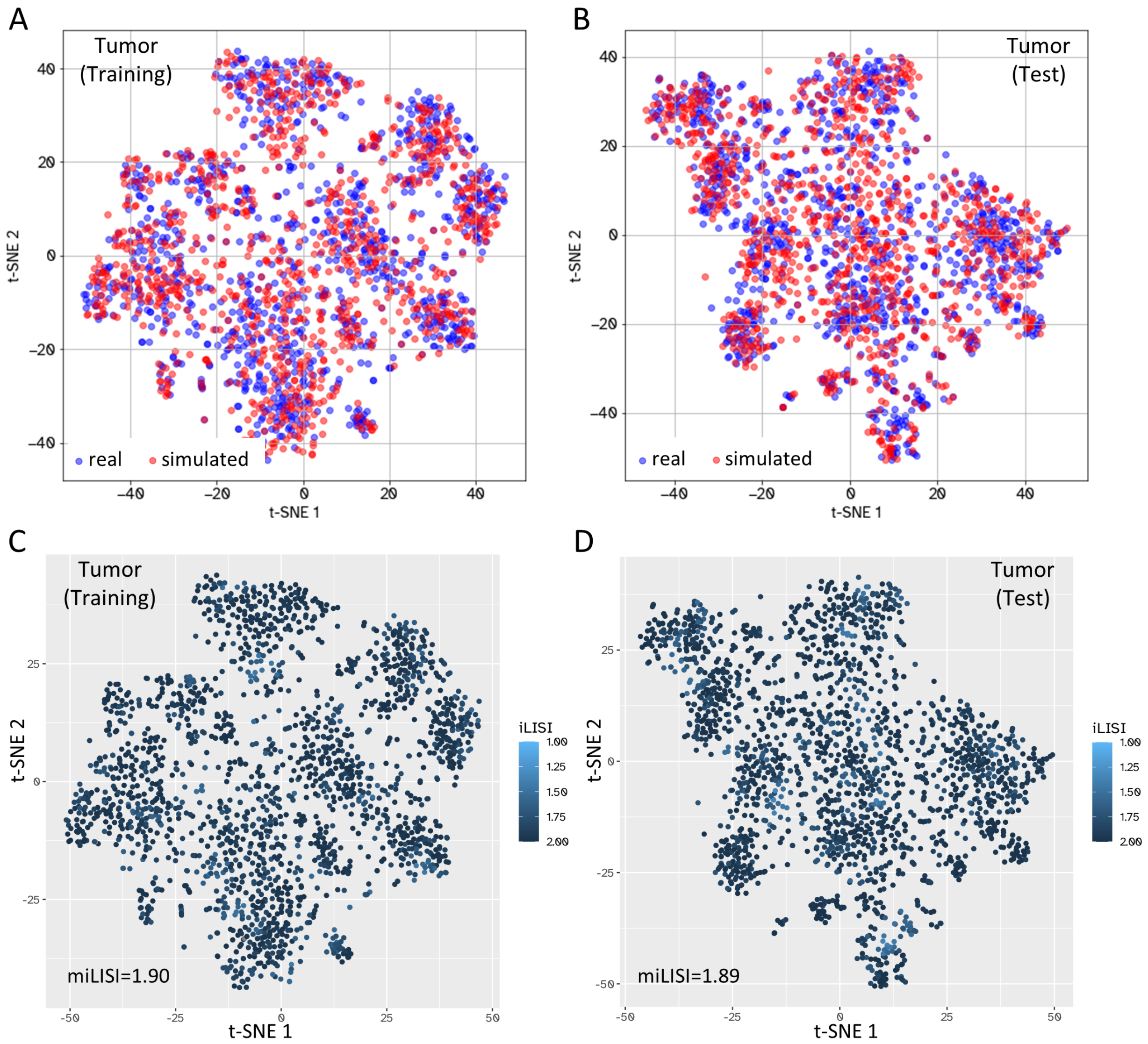

Supplementary Fig. 20: Real (experimental) and GRouNdGAN-simulated scRNA-seq data based on the Tumor-malignant dataset. All plots correspond to 1000 simulated cells and 1000 real cells. Each gene in the GRN of GRouNdGAN is regulated by 15 TFs (identified using GRNBoost2 from the experimental training dataset). Panels A and B show t-SNE plots of simulated cells (red) and real cells (blue). Panels C and D show the iLISI values of each datapoint and the average iLISI score of the data (miLISI). Panels A and C correspond to comparison between simulated cells and a random set of real cells in the training set, while panels B and D correspond to comparison between simulated cells and all the real cells in the test set. Source data are provided as a Source Data file.

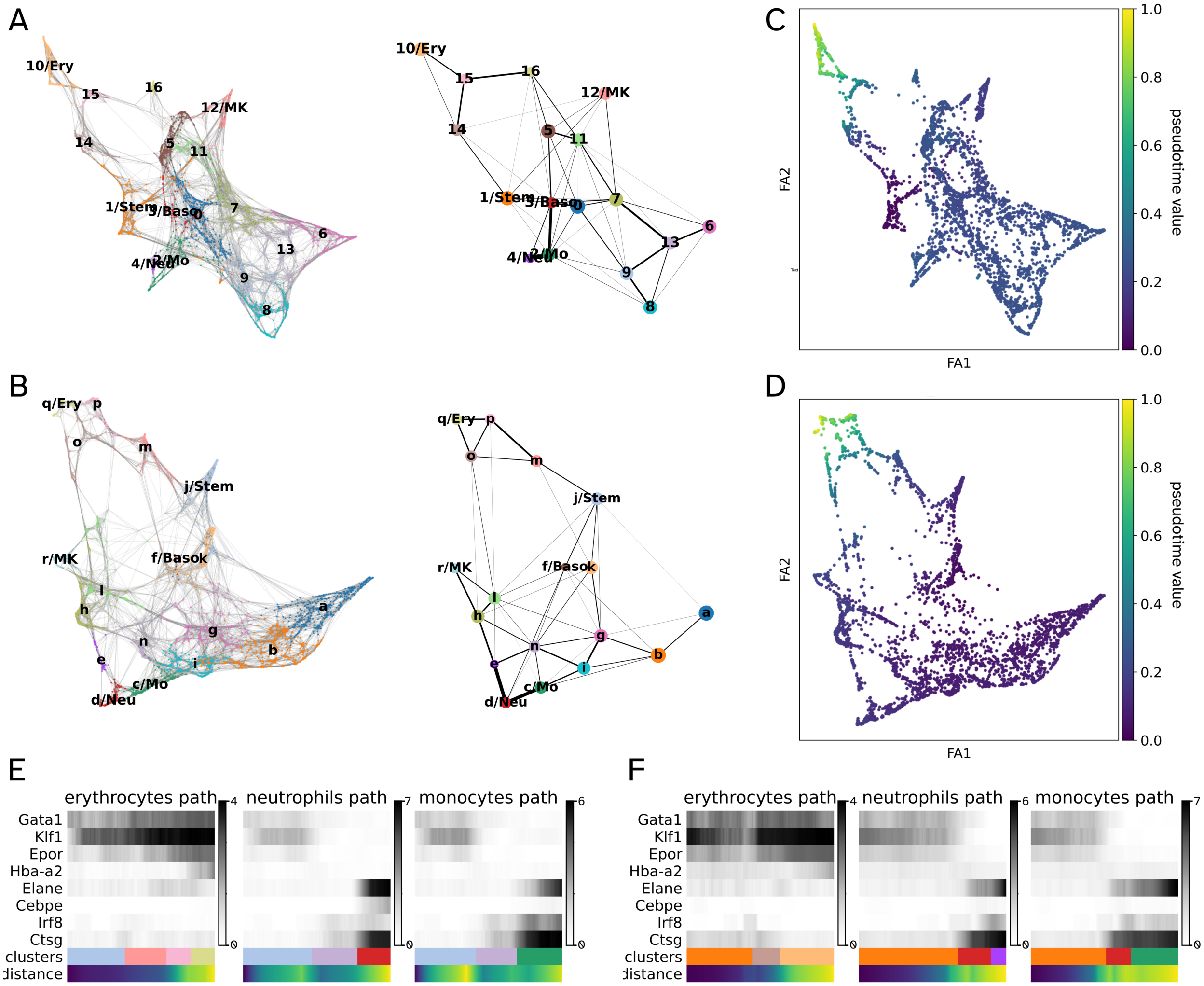

Supplementary Fig. 21: Consistency in trajectories and pseudo-temporal orderings between data generated by GRouNdGAN and the experimental Dahlin hematopoietic dataset. Panels A and B show the cell type annotations of simulated and experimental data, respectively. The figures on the left show the force-directed graphs (obtained using ForceAtlas2) and the figures on the right show the PAGA graphs. Panels C and D show the PAGA-initialized force-directed graphs with cells colored by their inferred pseudo-time value. The following abbreviations are used: Stem for stem cells, Ery for erythroid cells, Neu for neutrophils, Mo for monocytes, MK for Megakaryocytes, and Baso for Basophils. Panels E and F display expression changes in the marker genes of erythrocyte (*Gata1*, *Klf1*, Epor, and *Hba-a2*), neutrophil (*Elane* and *Cebpe*), and monocyte (*Irf8* and *Ctsg*) branches for experimental (E) and simulated data (F). The distance refers to the geodesic distance from a root cell (here a stem cell). A darker shade of grey shows higher expression. Source data are provided as a Source Data file.

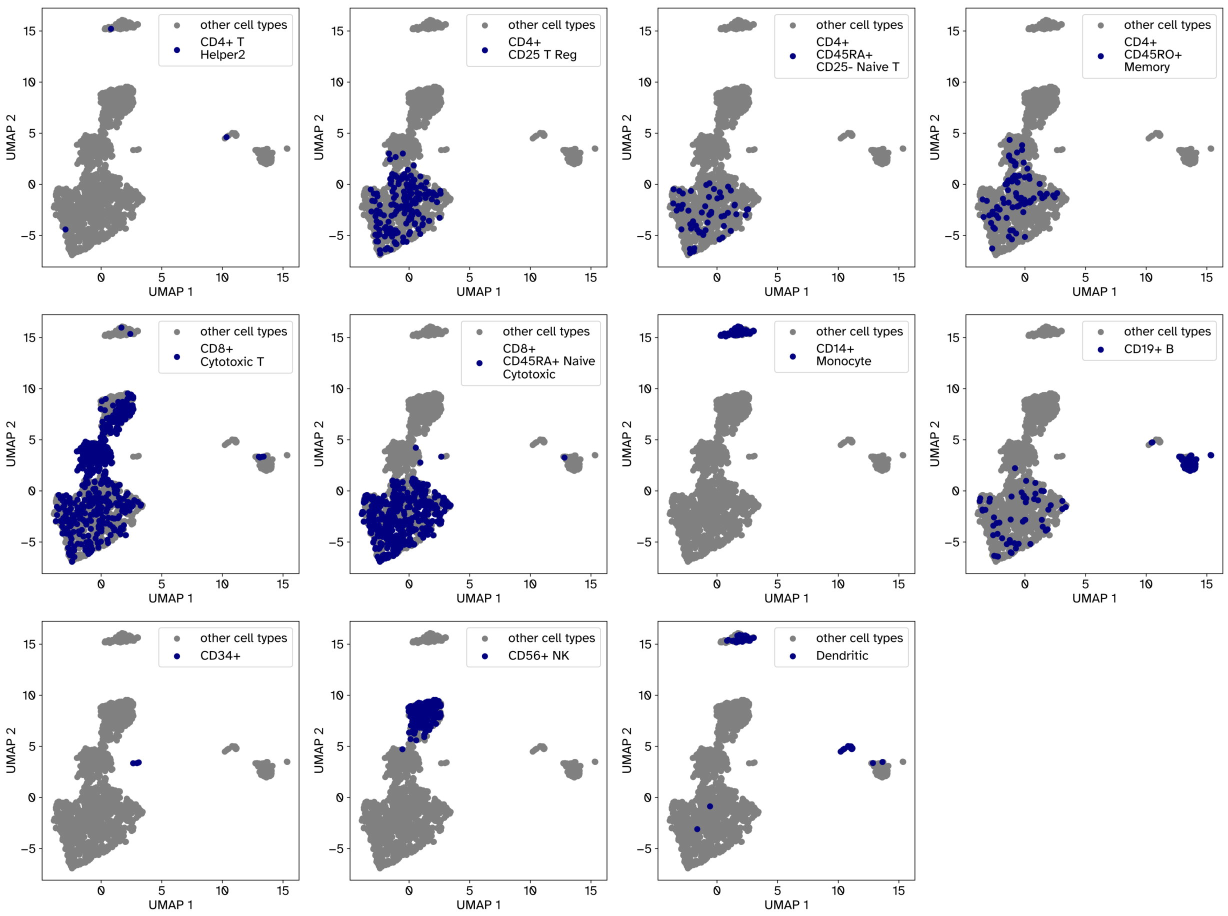

Supplementary Fig. 22: UMAP embedding of different cell types of the experimental PBMC-All dataset used for the analysis reported in Figure 6. We used cell-type annotations provided by 10x Genomics assigned by maximum correlation of PBMCs to filtered populations. Source data are provided as a Source Data file.

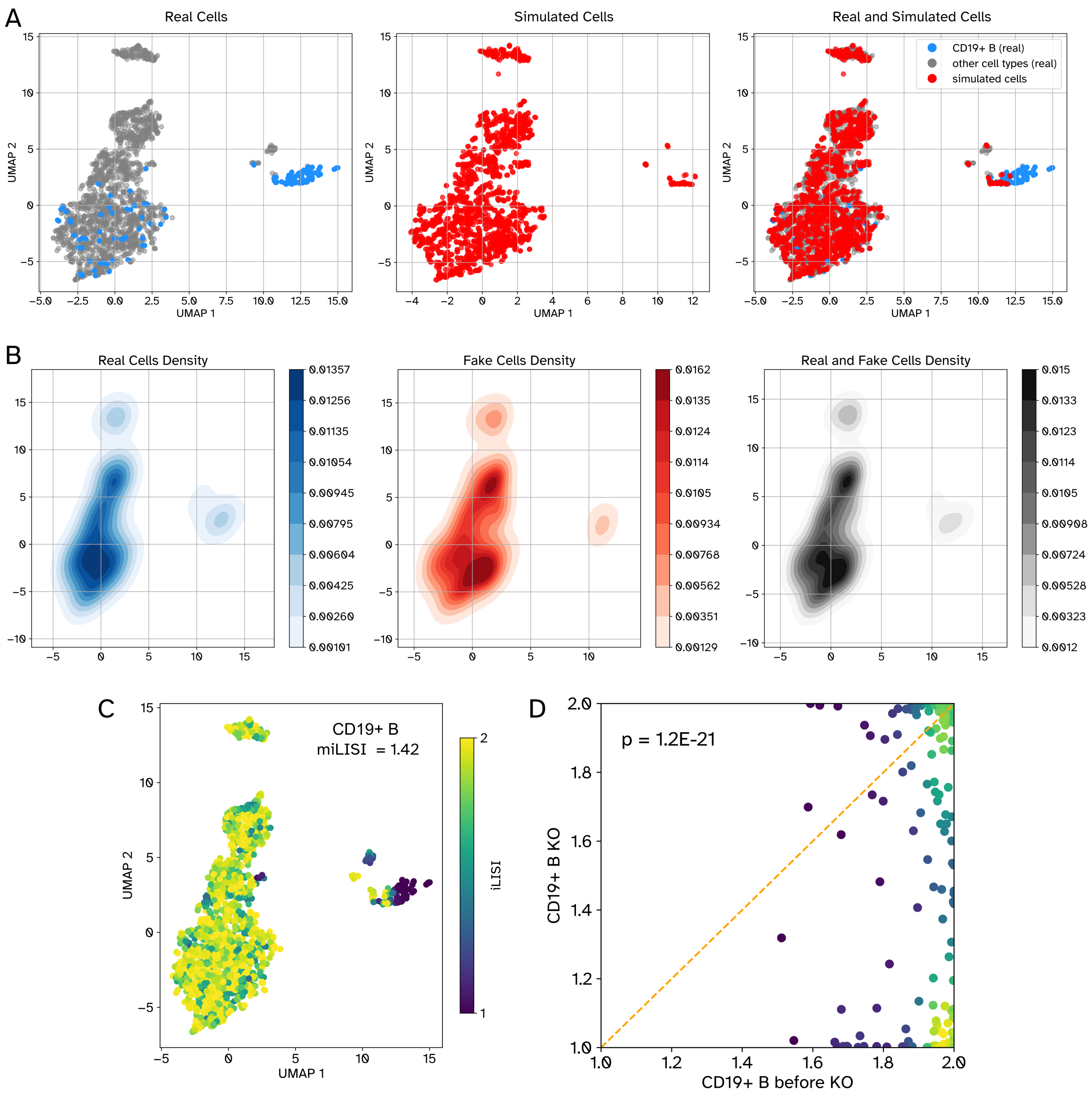

Supplementary Fig. 23: TF knockout analysis of CD 19+ B cells. Panels A and B show the UMAP and density plots of n = 2000 randomly selected cells from the experimental PBMC-All dataset (left), same number of simulated cells after knockout of top three TFs of CD19+ B cells (middle), and all together (right), respectively. TFs were omitted as features when generating UMAP plots. Panel C shows the iLISI value of each real cell (miLISI = 1.42) calculated from a UMAP embedding, jointly obtained from the experimental cells and the same number of simulated cells after knockout. D) The scatter plot shows the iLISI values of CD19+ B cells calculated along with unperturbed simulated cells (x-axis) and along with perturbed simulated cells (y-axis). The circles correspond to real cells and their colors reflect the density of datapoints in that region. Source data are provided as a Source Data file.

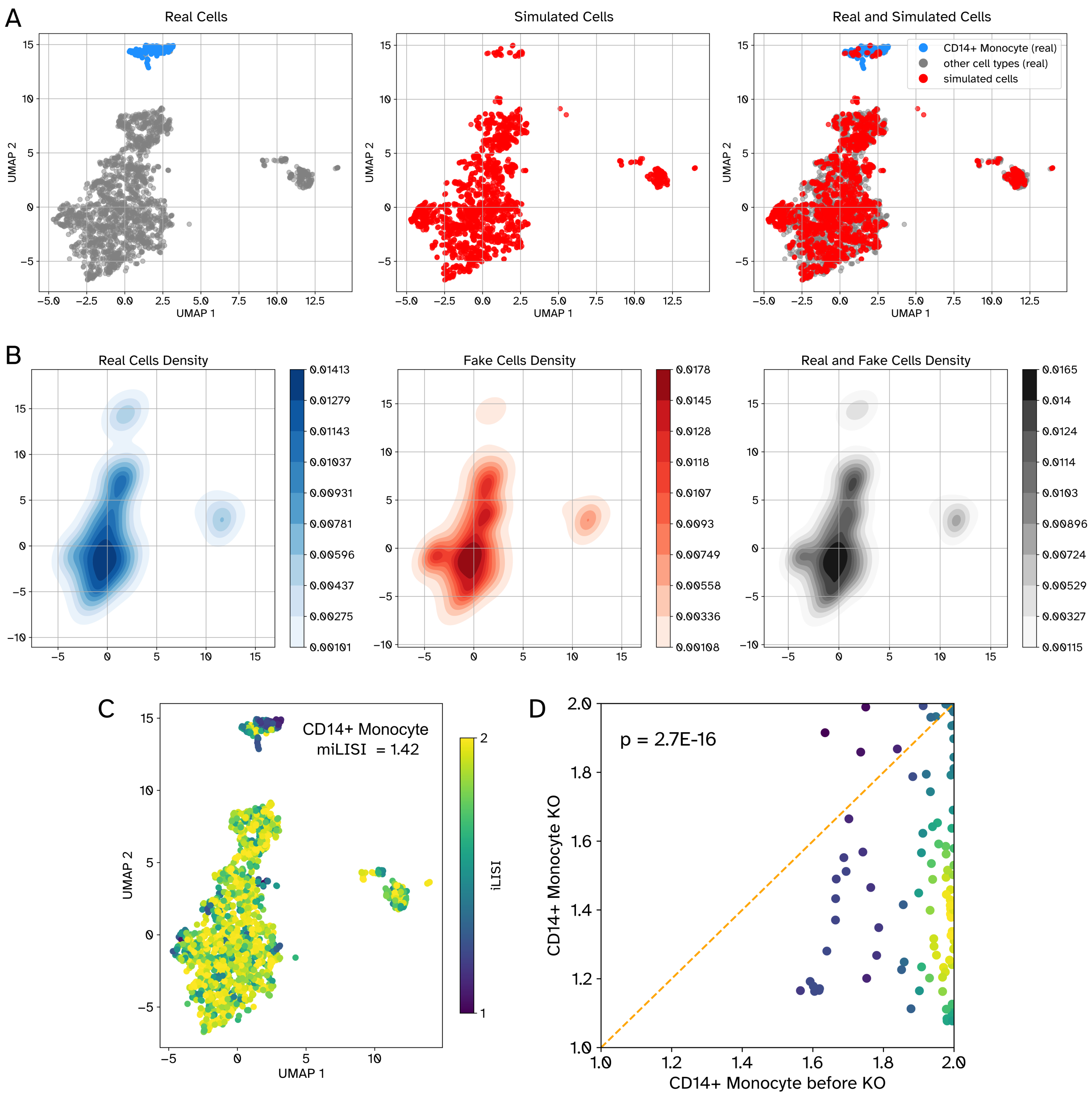

Supplementary Fig. 24: TF knockout analysis of CD14+ Monocyte cells. Panels A and B show the UMAP and density plots of 2000 randomly selected cells from the experimental PBMC-All dataset (left), same number of simulated cells after knockout of top three TFs of CD14+ Monocyte cells (middle), and all together (right), respectively. TFs were omitted as features when generating UMAP plots. Panel C shows the iLISI value of each real cell (miLISI = 1.42) calculated from a UMAP embedding, jointly obtained from the experimental cells and the same number of simulated cells after knockout. D) The scatter plot shows the iLISI values of CD14+ Monocyte cells calculated along with unperturbed simulated cells (x-axis) and along with perturbed simulated cells (y-axis). The circles correspond to real cells and their colors reflect the density of datapoints in that region. Source data are provided as a Source Data file.

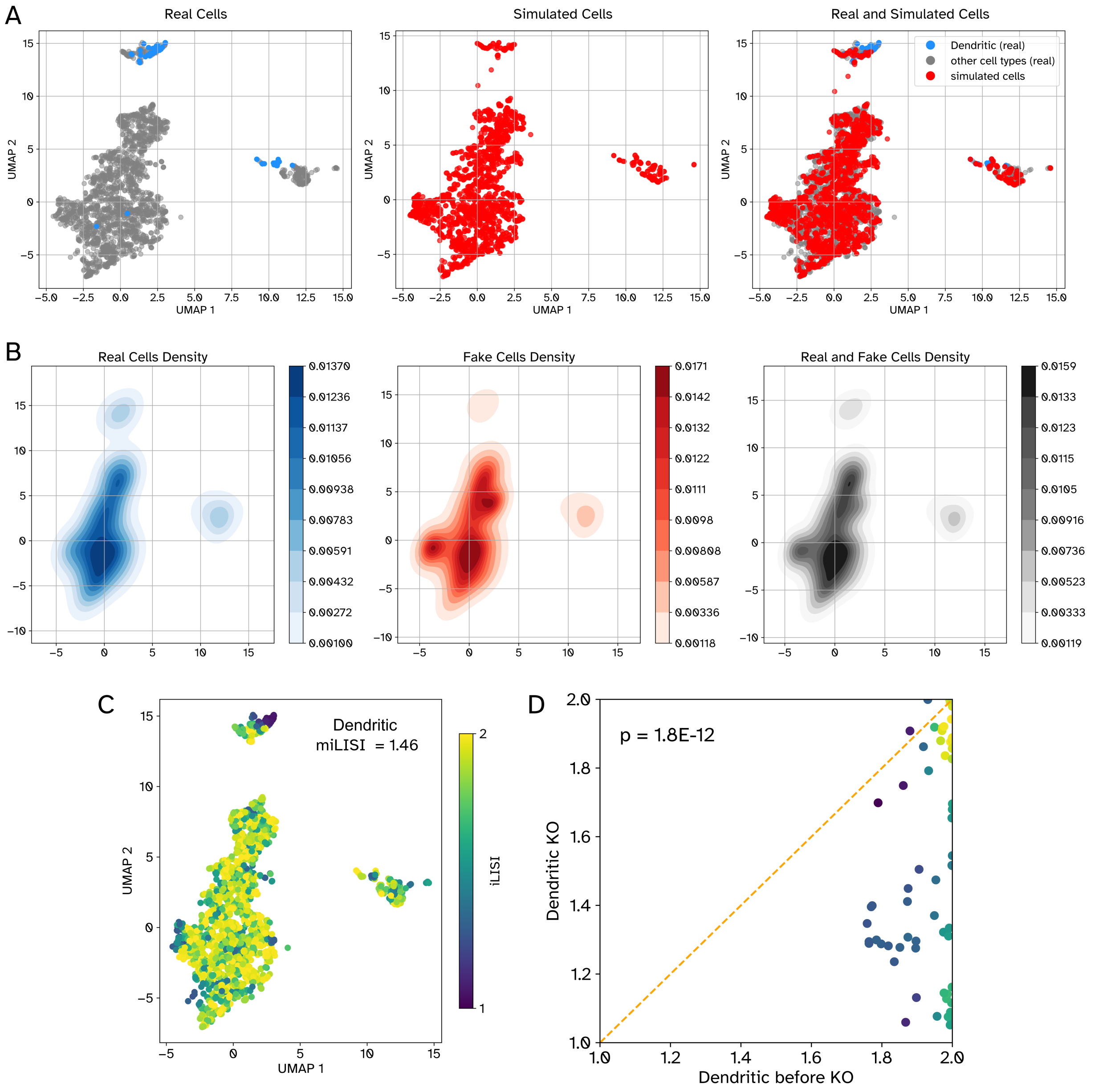

Supplementary Fig. 25: TF knockout analysis of Dendritic cells. Panels A and B show the UMAP and density plots of 2000 randomly selected cells from the experimental PBMC-All dataset (left), same number of simulated cells after knockout of top three TFs of Dendritic cells (middle), and all together (right), respectively. TFs were omitted as features when generating UMAP plots. Panel C shows the iLISI value of each real cell (miLISI = 1.46) calculated from a UMAP embedding, jointly obtained from the experimental cells and the same number of simulated cells after knockout. D) The scatter plot shows the iLISI values of Dendritic cells calculated along with unperturbed simulated cells (x-axis) and along with perturbed simulated cells (y-axis). The circles correspond to real cells and their colors reflect the density of datapoints in that region. Source data are provided as a Source Data file.

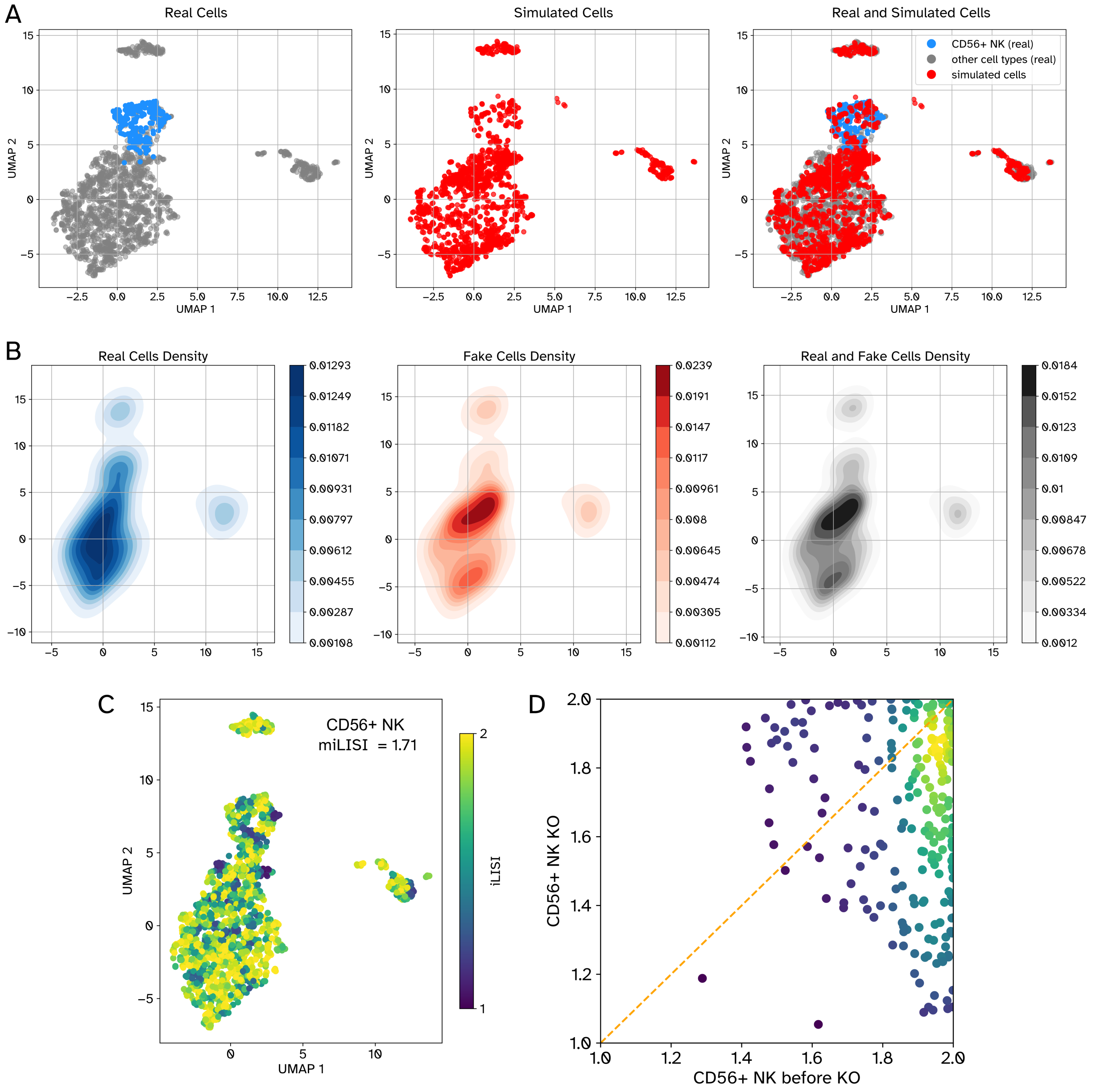

Supplementary Fig. 26: TF knockout analysis of CD56+ NK cells. Panels A and B show the UMAP and density plots of 2000 randomly selected cells from the experimental PBMC-All dataset (left), same number of simulated cells after knockout of top three TFs of CD56+ NK cells (middle), and all together (right), respectively. TFs were omitted as features when generating UMAP plots. Panel C shows the iLISI value of each real cell (miLISI = 1.71) calculated from a UMAP embedding, jointly obtained from the experimental cells and the same number of simulated cells after knockout. D) The scatter plot shows the iLISI values of CD56+ NK cells calculated along with unperturbed simulated cells (x-axis) and along with perturbed simulated cells (y-axis). The circles correspond to real cells and their colors reflect the density of datapoints in that region. Source data are provided as a Source Data file.

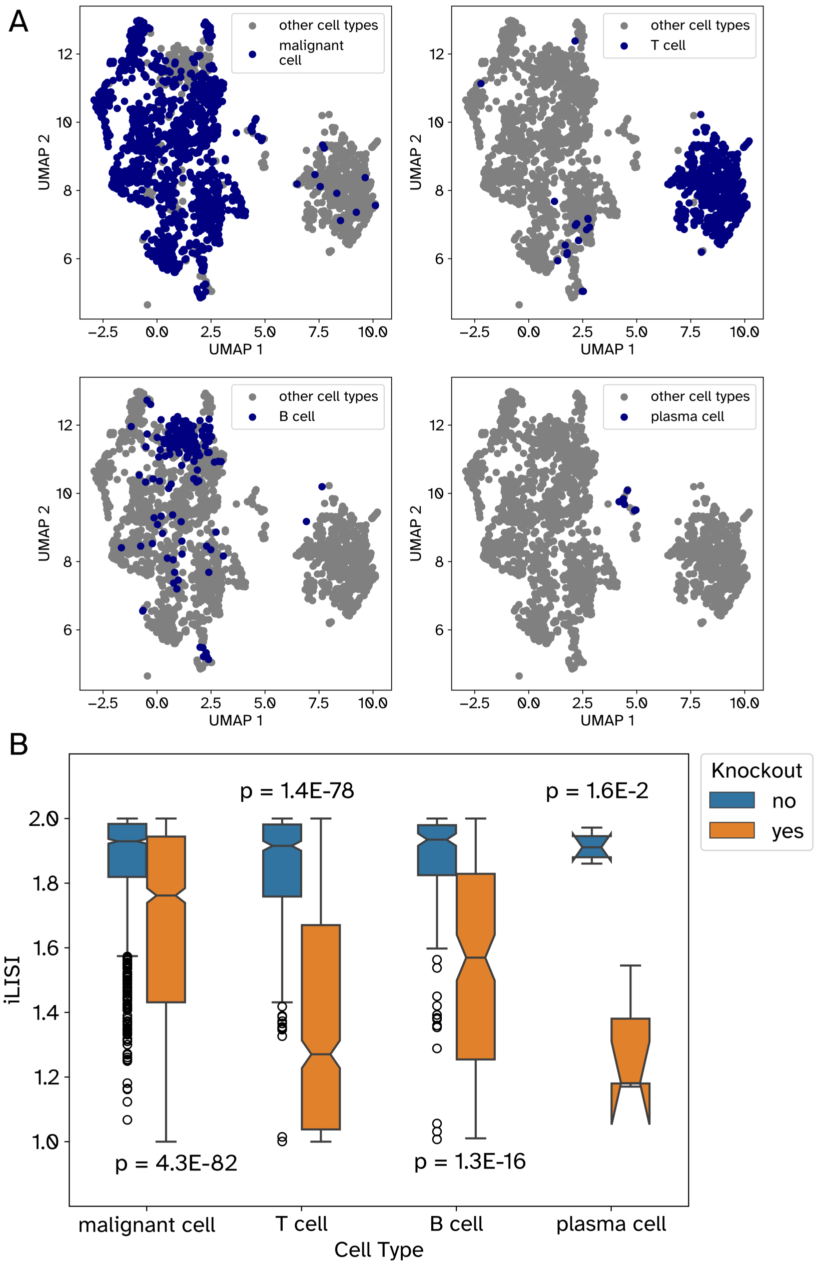

Supplementary Fig. 27: TF knockout analysis of four cell types in the Tumor-All dataset, based on 2000 randomly selected real cells and 2000 simulated cells. In the simulated data, each gene was regulated by 15 TFs, identified using GRNBoost2. We used cell-type annotations provided by the original study. Different subclasses of T Cells were merged as one cell type. A) UMAP embeddings of four cell types used for the analysis. B) The boxplots show the distribution of iLISI values of real cells, calculated along with unperturbed simulated cells (blue) and with perturbed simulated cells (orange). For each cell type, top three most differentially expressed TFs were knocked out. The p-values reported in this figure were calculated using one-sided Wilcoxon signed rank tests. Source data are provided as a Source Data file.

Supplementary References

1 Che, T., Li, Y., Jacob, A. P., Bengio, Y. & Li, W. Mode regularized generative adversarial networks. *arXiv preprint arXiv:1612.02136* (2016).

2 Gulrajani, I., Ahmed, F., Arjovsky, M., Dumoulin, V. & Courville, A. C. Improved training of wasserstein gans. *Advances in neural information processing systems* **30** (2017).

3 Arjovsky, M. & Bottou, L. Towards principled methods for training generative adversarial networks. *arXiv preprint arXiv:1701.04862* (2017).

4 Kushwaha, V. & Nandi, G. Study of prevention of mode collapse in generative adversarial network (GAN). In *2020 IEEE 4th Conference on Information & Communication Technology (CICT).* 1-6 (IEEE, 2020).

5 Liu, K., Tang, W., Zhou, F. & Qiu, G. Spectral regularization for combating mode collapse in GANs. In *Proceedings of the IEEE/CVF international conference on computer vision.* 6382-6390 (2019).

6 Yao, Y., Pan, Y., Tsang, I. W. & Yao, X. Support Matching: A Novel Regularization to Escape from Mode Collapse in GANs. In *International Conference on Neural Information Processing.* 40-48 (Springer, 2019).

7 Arjovsky, M., Chintala, S. & Bottou, L. Wasserstein generative adversarial networks. In *International conference on machine learning.* 214-223 (PMLR, 2017).

8 Villani, C. *Optimal transport: old and new*. Vol. 338 (Springer, 2009).

9 Wolf, F. A. *et al.* PAGA: graph abstraction reconciles clustering with trajectory inference through a topology preserving map of single cells. *Genome biology* **20**, 1-9 (2019).
