# Supplementary material for: GRouNdGAN: GRN-guided simulation of single-cell RNA-seq data using causal generative adversarial networks
Source: Nat Commun. 2024 May 14;15:4055. doi: 10.1038/s41467-024-48516-6 (PMC11525796; doi:10.1038/s41467-024-48516-6)
Supplement: Supplementary file 3 — Description of Additional Supplementary Files [file 41467_2024_48516_MOESM3_ESM.pdf]

## Description of Additional Supplementary Files:

**Supplementary Data 1:** The performance of di?erent simulators in generating realistic simulated data using three datasets. The table is provided as a separate xlsx file.

**Supplementary Data 2:** Stability analysis and the e?ect of di?erent GRN properties on the performance. The table is provided as a separate xlsx file.

**Supplementary Data 3:** Results of GRN inference using di?erent datasets simulated by GrouNdGAN. The table is provided as a separate xlsx file.
