# Supplementary material for: GRouNdGAN: GRN-guided simulation of single-cell RNA-seq data using causal generative adversarial networks
Source: Nat Commun. 2024 May 14;15:4055. doi: 10.1038/s41467-024-48516-6 (PMC11525796; doi:10.1038/s41467-024-48516-6)
Supplement: Supplementary file 7 — Reporting Summary [file 41467_2024_48516_MOESM7_ESM.pdf]

nature portfolio

Corresponding author(s): Amin Emad

Last updated by author(s): Apr 4, 2024

Reporting Summary

Nature Portfolio wishes to improve the reproducibility of the work that we publish. This form provides structure for consistency and transparency in reporting. For further information on Nature Portfolio policies, see our Editorial Policies and the Editorial Policy Checklist.

Statistics

For all statistical analyses, confirm that the following items are present in the figure legend, table legend, main text, or Methods section.

n/a	Confirmed	
☐	☒	The exact sample size (*n*) for each experimental group/condition, given as a discrete number and unit of measurement
☐	☒	A statement on whether measurements were taken from distinct samples or whether the same sample was measured repeatedly
☐	☒	The statistical test(s) used AND whether they are one- or two-sided<br>*Only common tests should be described solely by name; describe more complex techniques in the Methods section.*
☒	☐	A description of all covariates tested
☐	☒	A description of any assumptions or corrections, such as tests of normality and adjustment for multiple comparisons
☒	☐	A full description of the statistical parameters including central tendency (e.g. means) or other basic estimates (e.g. regression coefficient) AND variation (e.g. standard deviation) or associated estimates of uncertainty (e.g. confidence intervals)
☐	☒	For null hypothesis testing, the test statistic (e.g. F, t, r) with confidence intervals, effect sizes, degrees of freedom and P value noted<br>*Give P values as exact values whenever suitable.*
☒	☐	For Bayesian analysis, information on the choice of priors and Markov chain Monte Carlo settings
☒	☐	For hierarchical and complex designs, identification of the appropriate level for tests and full reporting of outcomes
☒	☐	Estimates of effect sizes (e.g. Cohen's *d*, Pearson's *r*), indicating how they were calculated

Our web collection on statistics for biologists contains articles on many of the points above.

Software and code

Policy information about availability of computer code

Data collection	No software was used for data collection. Details of how data was downloaded is described in the manuscript (see Data Availability section).
Data analysis	GRouNDGAN's implementation in Python 3.9.6 using the PyTorch framework is freely available at https://github.com/Emad-COMBINE-lab/GRouNdGAN.<br><br>Python dependencies: Software dependencies for reproducing a virtual environment are listed in the requirements.txt file provided in the repository.<br><br>R dependencies: We used R version 4.2.1 to run scDESIGN2, SPARSIM, and LISI (version: 1.0, SHA: a917556).<br><br>Included studies: BEELINE, scGAN, SPARSim, and scDESIGN2 are included as git submodules in our repository. This creates a snapshot indicating their commit SHAs (scGAN:988ad95, BEELINE: 79775f0, scDESIGN2 version 1.0.0: 4b5c819, SPARSim version 0.9.5: 0ac17a8).<br><br>GRN inference algorithms: Please refer to BEELINE's Supplementary Table 9, which details the specific version (or git commit hash) of GRN inference algorithms benchmarked in this study (PIDC, GENIE3, GRNBoost2, PPCOR, LEAP, SCODE, and SINCERITIES). For CeSGRN, we used the most recent commit (SHA: 7e56ce8).

For manuscripts utilizing custom algorithms or software that are central to the research but not yet described in published literature, software must be made available to editors and reviewers. We strongly encourage code deposition in a community repository (e.g. GitHub). See the Nature Portfolio guidelines for submitting code & software for further information.

Data

Policy information about availability of data

All manuscripts must include a data availability statement. This statement should provide the following information, where applicable:

- Accession codes, unique identifiers, or web links for publicly available datasets
- A description of any restrictions on data availability
- For clinical datasets or third party data, please ensure that the statement adheres to our policy

A data availability section is included in the manuscript. All datasets used in this study are publicly available. Accession codes and links for downloading the datasets are given in the manuscript.

The PMBC dataset is available from the 10x genomics repository (corresponding to healthy donor A) from the link "https://support.10xgenomics.com/single-cell-gene-expression/datasets/1.1.0/fresh_68k_pbmc_donor_a?". The BoneMarrow dataset is available in the Gene Expression Omnibus (GEO) repository under accession number GSE72857. The Dahlin dataset is available on GEO (accession number: GSE107727). The Tumor-ALL dataset can be found on cellxgene: https://cellxgene.cziscience.com/collections/968834a0-1895-40df-8720-666029b3bbac.

A collection of simulated datasets with known ground truth GRNs are provided on GRouNdGAN's website (https://emad-combine-lab.github.io/GRouNdGAN/) to enable GRN inference benchmarking on various datasets.

Research involving human participants, their data, or biological material

Policy information about studies with human participants or human data. See also policy information about sex, gender (identity/presentation), and sexual orientation and race, ethnicity and racism.

Reporting on sex and gender	Not applicable.
Reporting on race, ethnicity, or other socially relevant groupings	Not applicable.
Population characteristics	Not applicable.
Recruitment	Not applicable.
Ethics oversight	Not applicable.

Note that full information on the approval of the study protocol must also be provided in the manuscript.

Field-specific reporting

Please select the one below that is the best fit for your research. If you are not sure, read the appropriate sections before making your selection.

☒ Life sciences ☐ Behavioural & social sciences ☐ Ecological, evolutionary & environmental sciences

For a reference copy of the document with all sections, see nature.com/documents/nr-reporting-summary-flat.pdf

Life sciences study design

All studies must disclose on these points even when the disclosure is negative.

Sample size	Data was downloaded from existing (public) experimental datasets with sample sizes determined in their original publication (and mentioned in the manuscript). Simulated dataset sample sizes for different analyses are indicated in the manuscript. Datasets included were chosen in a way to cover diverse sample sizes and a broad range of cell types, conditions (e.g., tumor), and processes (e.g., differentiation).
Data exclusions	We filtered low-quality cells by removing cells with nonzero counts in less than ten genes. Likewise, genes expressed in less than three cells were discarded to reduce technical noise introduced by low-quality reads. We focused on the most informative genes by only studying the 1000 top highly variable ones. Other than this standard cell-level and gene-level filtering, there were no other data exclusions.
Replication	We validated our method, keeping the same hyperparameters on various GRN combinations with seven datasets, each having vastly different characteristics. We also performed a stability analysis which revealed similar results across multiple runs and models. Since we used published datasets, biological replicate analysis is not applicable.
Randomization	Randomization is not applicable to our study, as we use existing published datasets.
Blinding	Blinding is not applicable to our study, as it is describing a computational method. However, test sets not used for training were used to evaluate the model.

Reporting for specific materials, systems and methods

We require information from authors about some types of materials, experimental systems and methods used in many studies. Here, indicate whether each material, system or method listed is relevant to your study. If you are not sure if a list item applies to your research, read the appropriate section before selecting a response.

Materials & experimental systems

n/a	Involved in the study
☒	☐ Antibodies
☒	☐ Eukaryotic cell lines
☒	☐ Palaeontology and archaeology
☒	☐ Animals and other organisms
☒	☐ Clinical data
☒	☐ Dual use research of concern
☒	☐ Plants

Methods

n/a	Involved in the study
☒	☐ ChIP-seq
☒	☐ Flow cytometry
☒	☐ MRI-based neuroimaging

Plants

Seed stocks	*Report on the source of all seed stocks or other plant material used. If applicable, state the seed stock centre and catalogue number. If plant specimens were collected from the field, describe the collection location, date and sampling procedures.*
Novel plant genotypes	*Describe the methods by which all novel plant genotypes were produced. This includes those generated by transgenic approaches, gene editing, chemical/radiation-based mutagenesis and hybridization. For transgenic lines, describe the transformation method, the number of independent lines analyzed and the generation upon which experiments were performed. For gene-edited lines, describe the editor used, the endogenous sequence targeted for editing, the targeting guide RNA sequence (if applicable) and how the editor was applied.*
Authentication	*Describe any authentication procedures for each seed stock used or novel genotype generated. Describe any experiments used to assess the effect of a mutation and, where applicable, how potential secondary effects (e.g. second site T-DNA insertions, mosiacism, off-target gene editing) were examined.*
